# Prdm6 controls heart development by regulating neural crest cell differentiation and migration

**DOI:** 10.1172/jci.insight.156046

**Published:** 2022-02-22

**Authors:** Lingjuan Hong, Na Li, Victor Gasque, Sameet Mehta, Lupeng Ye, Yinyu Wu, Jinyu Li, Andreas Gewies, Jürgen Ruland, Karen K. Hirschi, Anne Eichmann, Caroline Hendry, David van Dijk, Arya Mani

**Affiliations:** 1 Cardiovascular Research Center, Department of Internal Medicine,; 2 Yale Center for Genome Analysis, and; 3 Department of Genetics, Yale University School of Medicine, New Haven, Connecticut, USA.; 4 Helmholtz Zentrum München, Munich, Germany.; 5 The Technical University of Munich, Munich, Germany.; 6 University of Virginia School of Medicine, Charlottesville, Virginia, USA.; 7 Department of Cellular and Molecular Physiology, Yale University School of Medicine, New Haven, Connecticut, USA.; 8 Yale Department of Computer Science, Yale University, New Haven, Connecticut, USA.

**Keywords:** Cardiology, Development, Cardiovascular disease, Embryonic development, Epigenetics

## Abstract

The molecular mechanisms that drive the acquisition of distinct neural crest cell (NCC) fates is still poorly understood. Here, we identified Prdm6 as an epigenetic modifier that temporally and spatially regulates the expression of NCC specifiers and determines the fate of a subset of migrating cardiac NCCs (CNCCs). Using transcriptomic analysis and genetic and fate mapping approaches in transgenic mice, we showed that disruption of *Prdm6* was associated with impaired CNCC differentiation, delamination, and migration and led to patent ductus arteriosus (DA) and ventricular noncompaction. Bulk and single-cell RNA-Seq analyses of the DA and CNCCs identified *Prdm6* as a regulator of a network of CNCC specification genes, including *Wnt1*, *Tfap2b*, and *Sox9*. Loss of Prdm6 in CNCCs diminished its expression in the pre-epithelial–mesenchymal transition (pre-EMT) cluster, resulting in the retention of NCCs in the dorsal neural tube. This defect was associated with diminished H4K20 monomethylation and G1-S progression and augmented *Wnt1* transcript levels in pre-EMT and neural tube clusters, which we showed was the major driver of the impaired CNCC migration. Altogether, these findings revealed *Prdm6* as a key regulator of CNCC differentiation and migration and identified *Prdm6* and its regulated network as potential targets for the treatment of congenital heart diseases.

## Graphical abstract

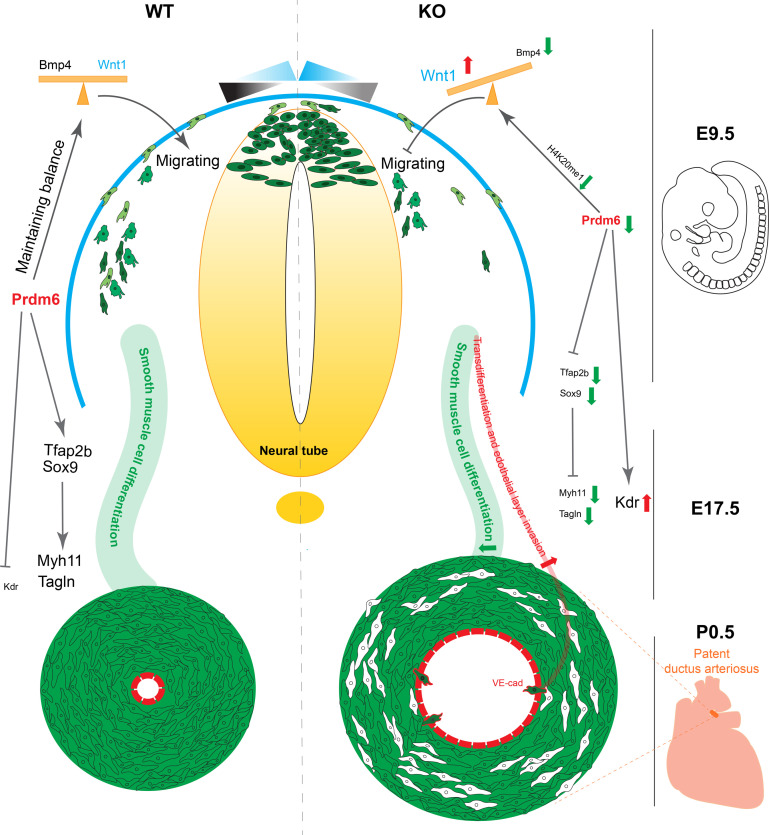

## Introduction

Neural crest cells (NCCs) are a transient population of multipotent cells unique to vertebrates that arise from neural folds during embryonic development (1) and migrate throughout the body, giving rise to diverse cell lineages. In mice, cardiac NCCs (CNCCs) — precursors to the heart — are generated at 8–8.5 dpc and arrive in the distal outflow at E9–9.5 (2). Targeted ablation of *Wnt1*-expressing CNCCs at 8.5 dpc in mice results in a complex phenotype of craniofacial and cardiovascular outflow tract defects (3). Similarly, the impaired function of CNCCs in humans underlies the pathogenesis of various complex human congenital disorders collectively known as cardio craniofacial syndromes (4). Cell lineage analyses have helped uncover the spatial and temporal diversification of NCC lineages (5, 6). However, the mechanisms underlying the pleiotropic effects of CNCCs are not understood owing to a lack of insight into how subsets of CNCCs are specified and how their subsequent fate is determined.

PRDM6 is a smooth muscle cell–specific (SMC-specific) histone methyltransferase and a member of the PRDM family of transcriptional repressors. It is expressed in the cardiac outflow tract and the ductus arteriosus (DA), a small artery that connects the aorta and pulmonary artery (7). Mice globally deficient for *Prdm6* are embryonically lethal because of abnormal vascular patterning (8). In humans, loss-of-function mutations in the *PRDM6* gene have been the first, and to this date, the only known genetic cause of the familial nonsyndromic patent DA (PDA) (9). PDA is an extreme example of an isolated cardiac disease that results from failure of closure of the DA, a derivative of the sixth pharyngeal arch, which is largely derived from premigratory CNCCs (10). The tunica media of the DA and other pharyngeal arch–remodeled arteries consists mainly of neural crest–derived SMCs (11). Syndromic PDA is caused by mutation in the NCC-specifier *TFAP2B* (12, 13). However, whether a defect in NCCs underpins the nonsyndromic PDAs and whether *PRDM6* plays a causal role in this process have never been investigated. Here, we combined bulk and single-cell RNA-Seq (scRNA-Seq) with 2 mouse models and identified *Prdm6* as a hub that regulates a network of genes involved in CNCC specification and orchestrates the delamination, migration, and differentiation of a subgroup of CNCCs required for DA formation in a Wnt1-dependent manner.

## Results

### Prdm6 expression in CNCCs is required for embryonic heart development.

**Figure 1:**
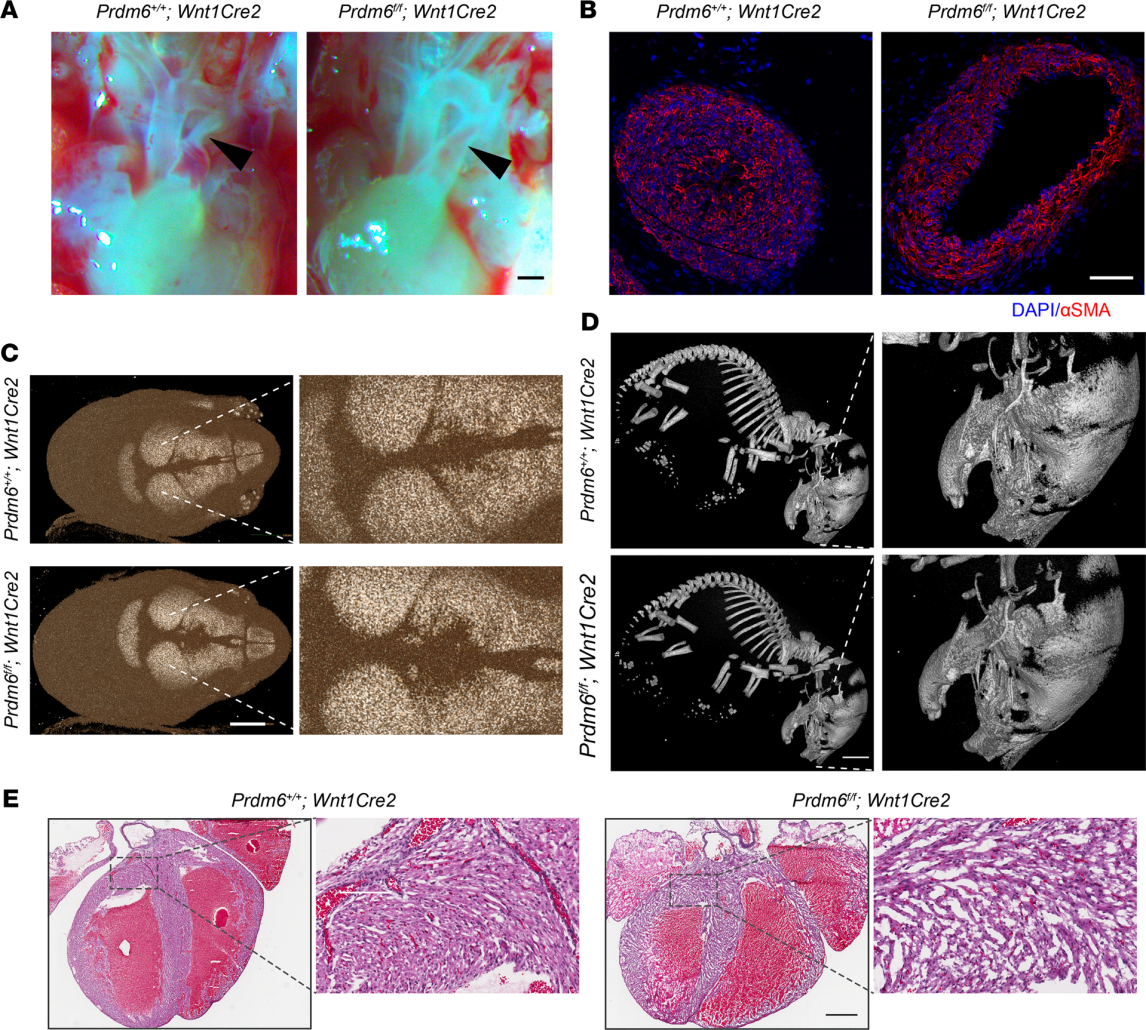
NCC-specific *Prdm6*-KO mice are born with PDA and noncompacted myocardium. (**A**) The gross appearance of a control heart (left) and *Prdm6^fl/fl^ Wnt1-Cre2* heart (right) and great cardiac vessels at P0.5. The closed DA and patent DA are shown by black arrowheads. Scale bar: 200 μm. (**B**) The confocal images of representative cross-sections of the DA of control (left) and *Prdm6^fl/fl^ Wnt1-Cre2* (right) pups at P0.5 stained with antibodies against αSMA (red) and DAPI (blue). Scale bar: 100 μm. (**C**) The computer micrographs of control (top) and *Prdm6^fl/fl^ Wnt1-Cre2* (bottom) pups’ skulls at P0.5, demonstrating small defect in the frontal bone at the junction of the anterior fontanel from apical views. Scale bar: 2 mm. The right panels show the same images magnified for better visualization. (**D**) The computer micrographs of control (top) and *Prdm6^fl/fl^ Wnt1-Cre2* (bottom) pups’ skeletons and skulls at P0.5 from lateral views. Scale bar: 3 mm. (**E**) H&E staining of control (left) and *Prdm6^fl/fl^ Wnt1Cre2* (right) pup hearts, demonstrating biventricular noncompaction but no other gross structural anomalies. Scale bar: 500 μm. All controls were the corresponding littermates (*n* = 3–6 for each group).

NCCs contribute to the development of the sixth aortic arch and its derivatives, the DA and pulmonary artery, and give rise to the tunica media of these arteries (14). To determine whether *Prdm6* is involved in the specification of NCCs and formation of the DA, we crossbred *Prdm6^fl/fl^ and Wnt1-Cre2* mice for several generations to generate *Prdm6^fl/fl^ Wnt1-Cre2* mice. According to an earlier study, *Wnt1* expression in CNCCs starts on dpc 8–8.5 (15), while *Prdm6* expression has been shown to be readily detectable in the embryo by E9.5 using Northern blot analysis (8). No gross syndromic abnormalities were noted in *Prdm6^fl/fl^ Wnt1-Cre2* mice (Supplemental Figure 1A; supplemental material available online with this article; https://doi.org/10.1172/jci.insight.156046DS1). *Prdm6^fl/fl^ Wnt1-Cre2* mice died on P0.5 with widely open DAs (Figure 1A). There was no abnormality of the great arteries or their branching (Figure 1A). The cross-section examination of DAs in *Prdm6^fl/fl^ Wnt1-Cre2* mice showed a large and widely patent lumen (Figure 1B). The examination of the skeleton of the *Prdm6^fl/fl^ Wnt1-Cre2* mice showed only a small defect in the frontal bone at the junction of the anterior fontanel (Figure 1C) and no other defects (Figure 1D). The examination of the heart showed biventricular noncompaction but no other structural defect (Figure 1E). These findings indicate that *Prdm6* expression in CNCCs is required for proper closure of the DA and the development of compacted myocardium during embryonic development. The cardiovascular traits were completely penetrant in both male and female mice (Table 1)

### Loss of Prdm6 diminishes the contribution of CNCCs to the myocardium and DA.

**Figure 2:**
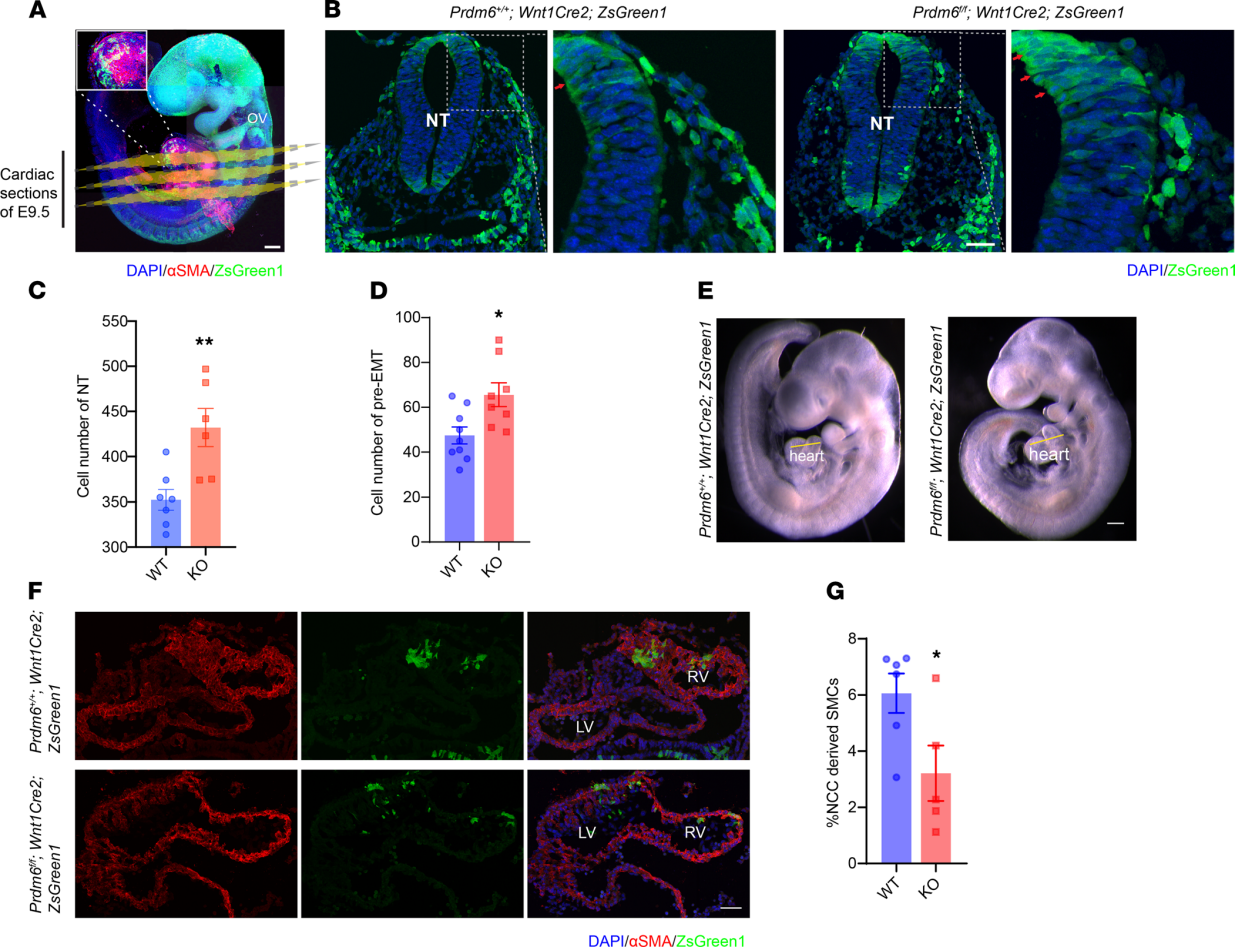
Fate mapping of CNCC establishes the role of *Prdm6* in CNCC migration. (**A**) Whole-mount confocal image of *Prdm6^+/+^ Wnt1-Cre2 ZsGreen1* E9.5 embryo. The inset shows the colocalization of αSMA (red) with ZsGreen1(green); nuclei are stained with DAPI (blue). Scale bar: 200 μm. (**B**) Transverse sections of cardiac neural crest at E9.5, using confocal imaging, demonstrating the retention of ZsGreen1-positive neural crest cells (green) in the neural tube (red arrow) in *Prdm6^fl/fl^ Wnt1-Cre2 ZsGreen1* versus littermate controls (*Prdm6^f+/+^ Wnt1-Cre2 ZsGreen1*); nuclei are stained with DAPI (blue), scale bar: 50 μm. (**C** and **D**) Quantification of total neural tube and pre-EMT cells, respectively. (**E** and **F**) Cross-sections of E9.5 myocardium using confocal imaging, showing thinner myocardium and lesser CNCC contribution to myocardium in *Prdm6^fl/fl^ Wnt1-Cre2 ZsGreen1* (bottom) versus controls (*Prdm6^f+/+^ Wnt1-Cre2 ZsGreen1,* top); αSMA (red), DAPI (blue), and ZsGreen1 (green). LV, left ventricle; RV, right ventricle. Scale bar: 50 μm. (**G**) Percentage of NCC-derived smooth muscle cells (SMCs) in myocardium. The image intensities were quantified by ImageJ; the thresholds for positive color detection were kept constant between different images. Each dot represents a biological replicate. The comparison between different groups was done by a 2-tailed unpaired *t* test, and data are shown as mean ± SEM. *P < 0.05, **P < 0.01. All controls were the corresponding littermates (*n* = 5–9 per group).

To determine how CNCC fate is affected by loss of *Prdm6*, we performed lineage tracing of *Wnt1*-CNCCs using *Wnt1-Cre2 ZsGreen1* mice (*Prdm6^+/+^*) and *Prdm6^fl/fl^ Wnt1-Cre2 ZsGreen1* mice in which *Prdm6* expression was disrupted specifically in CNCCs. In both cases, all *Wnt1*-CNCCs and their progeny were labeled with ZsGreen1 cell body (Figure 2A). The transverse sections of the cardiac neural crest in *Prdm6^fl/fl^ Wnt1-Cre2 ZsGreen1* embryos at E9.5 showed significant retention of ZsGreen1-positive cells in the neural tube (red arrows) and increased cell number in the neural tube compared with *Prdm6^+/+^ Wnt1-Cre2 ZsGreen1* (WT) mice, suggestive of impaired NCC delamination (Figure 2, B–D). Examination of the great vessels and semilunar valves at P0.5 revealed no significant difference between *Prdm6^fl/fl^ Wnt1-Cre2 ZsGreen1* mice and WT controls (Supplemental Figure 1B). However, the cross-sectioning of the heart at E9.5 showed reduced ZsGreen1-positive cells and thinning of the myocardium in *Prdm6^fl/fl^ Wnt1-Cre2 ZsGreen1* mice compared with WT mice (Figure 2, E–G).

**Figure 3:**
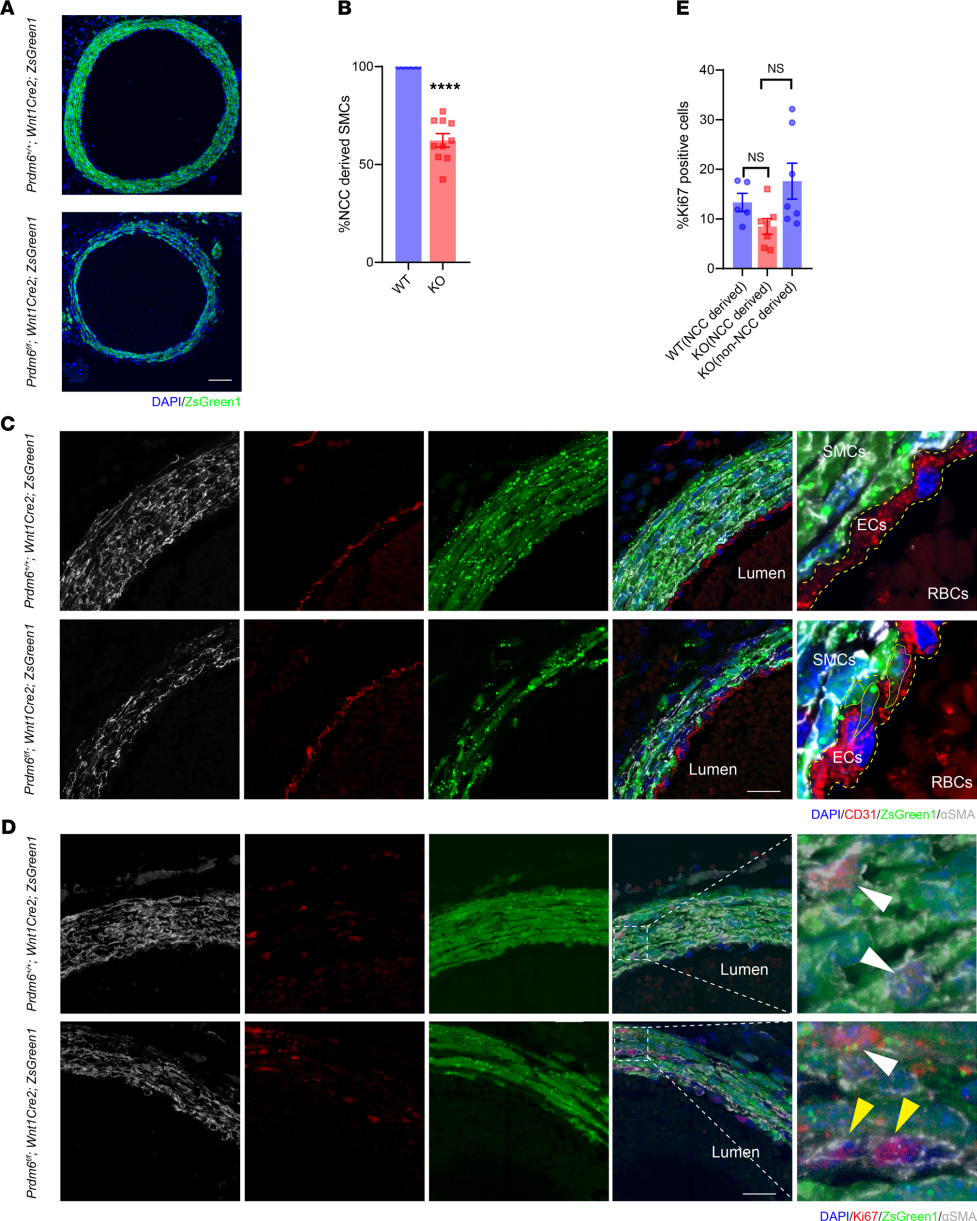
Fate mapping of CNCCs in neural crest–specific *Prdm6*-deficient mice shows their reduced contribution to ductus arteriosus. (**A**) Representative cross-sections of DA of WT (top) and *Prdm6^fl/fl^ Wnt1-Cre2 ZsGreen1* (bottom) DA at E17.5 by confocal imaging; scale bar: 50 μm. (**B**) Percentage of neural crest–derived SMCs. (**C**) Magnified image demonstrating the colocalization of αSMA (gray) and ZsGreen1 (green). In contrast to controls, the DA wall was thinner and only a fraction of SMCs of *Prdm6^fl/fl^ Wnt1-Cre2 ZsGreen1* were NCC derived. In addition, the NCC-derived SMCs of *Prdm6^fl/fl^ Wnt1-Cre2 ZsGreen1* have an irregular shape (outlined by straight yellow lines) and have infiltrated and disrupted the endothelial layer, stained with CD31 antibody (outlined by dotted yellow lines); scale bar: 20 μm. (**D**) The confocal images of representative cross-sections of DA of control (top row) and *Prdm6^fl/fl^ Wnt1-Cre2 ZsGreen1* (bottom row) mice at E17.5 stained for Ki67 (red) and αSMA (gray) show the localization of ZsGreen1 (green) and Ki67 (white arrowheads) and Ki67 in ZsGreen1-negative SMCs, indicated by yellow arrowheads. Scale bar: 50 μm. (**E**) Percentage of Ki67-positive cells in NCC-derived SMCs in WT mice and NCC-derived and non-NCC-derived SMCs in *Prdm6^fl/fl^ Wnt1-Cre2 ZsGreen1* (KO) DA. All SMCs in WT mice were NCC derived. There was no significant difference in the percentage of Ki67-positive NCC-derived SMCs between WT and KO mice. The image intensities were quantified by ImageJ, and the thresholds for positive color detection were kept constant between different images. Each dot represents a biological replicate. The comparison between different groups was done by a 2-tailed unpaired *t* test, and data are shown as mean ± SEM. The comparisons between multiple groups (**E**) were done by 1-way ANOVA. A Mann-Whitney test was conducted for non-normally distributed data. ****P < 0.0001. All controls were the corresponding littermates (*n* = 5–10 per group).

It has been shown that the SMCs in the tunica media of the DA are derived from CNCCs (16). The examination of the DA of *Prdm6^fl/fl^ Wnt1-Cre2 ZsGreen1* mice at E17.5 showed dramatically reduced ZsGreen1-positive (NCC-derived) cells in the tunica media compared with those of the WT littermates (Figure 3, A and B). In contrast to controls, the DA wall in *Prdm6^fl/fl^ Wnt1-Cre2 ZsGreen1* mice was thinner, and only a fraction of its SMCs was NCC-derived (Figure 3B). In addition, the NCC-derived SMCs of *Prdm6^fl/fl^ Wnt1-Cre2 ZsGreen1* mice had an irregular shape (Figure 3C) outlined by straight yellow lines and had infiltrated and disrupted the endothelial layer, stained with CD31 antibody and outlined by dotted yellow lines (Figure 3C). Endothelial cells of the DA are not NCC derived and undergo apoptosis at the onset of ductus closure (17), as shown in the WT ductus (Supplemental Figure 2A). In contrast, the endothelial layer of the patent ductus in *Prdm6^fl/fl^ Wnt1-Cre2 ZsGreen1* mice appeared intact at birth. Unexpectedly, we noted the costaining of ZsGreen1 and VE-cadherin in several cells within the ductus endothelial layers (Supplemental Figure 2, A and B) and fewer NCC-derived SMCs (Supplemental Figure 2C) in *Prdm6^fl/fl^ Wnt1-Cre2 ZsGreen1* mice versus WT littermates. These findings indicated the critical role of Prdm6 in NCC differentiation.

We then examined whether impaired proliferation of NCCs contributes to the failure of DA closure. We examined cell proliferation at E17.5 in *Prdm6^fl/fl^ Wnt1-Cre2 ZsGreen1* mice and their WT littermates using Ki67 staining (Figure 3D). We observed no significant difference in the percentage of Ki67-positive NCC-derived or non-NCC-derived SMCs between the 2 genotypes (Figure 3E). At birth, the DA cells are no longer proliferative and undergo apoptosis (18, 19). Accordingly, the Ki67 staining of the ductus in the WT mice showed absence of ductal cell proliferation (Supplemental Figure 2D). In contrast, ZsGreen1-negative and to a lesser degree ZsGreen1-positive ductal SMCs in *Prdm6^fl/fl^ Wnt1-Cre2 ZsGreen1* mice were Ki67 positive (Supplemental Figure 2E). Taken together, these findings indicated that the patency of the DA in *Prdm6^fl/fl^ Wnt1-Cre2 ZsGreen1* mice was not caused by impaired proliferation of NCC-derived SMCs. In addition, increased proliferation of non-NCC-derived SMCs appeared as insufficient to compensate for reduced NCC-derived SMCs in *Prdm6^fl/fl^ Wnt1-Cre2 ZsGreen1* mice. This finding is consistent with a prior report that NCC-derived and not mesodermal-derived SMCs are responsible for ductal closure (20).

In addition, numerous ZsGreen1-positive cells were detected in the ventricular septum and to a lesser degree in the innermost layer of the left and right ventricles of WT pups at P0.5. The number of ZsGreen1-positive cells in the myocardium of *Prdm6^fl/fl^ Wnt1-Cre2 ZsGreen1* mice was also significantly diminished compared with WT mice (Supplemental Figure 2, F and G), a finding that correlated with the ventricular noncompaction in these mice and suggested impaired NCC migration to the heart. Together, these results suggested that loss of *Prdm6* expression resulted in impaired NCC migration and differentiation and diminished their subsequent contribution to the DA and myocardium, prompting investigations into molecular mechanisms of the disease.

### Loss of Prdm6 impairs proper migration and differentiation of CNCCs.

**Figure 4:**
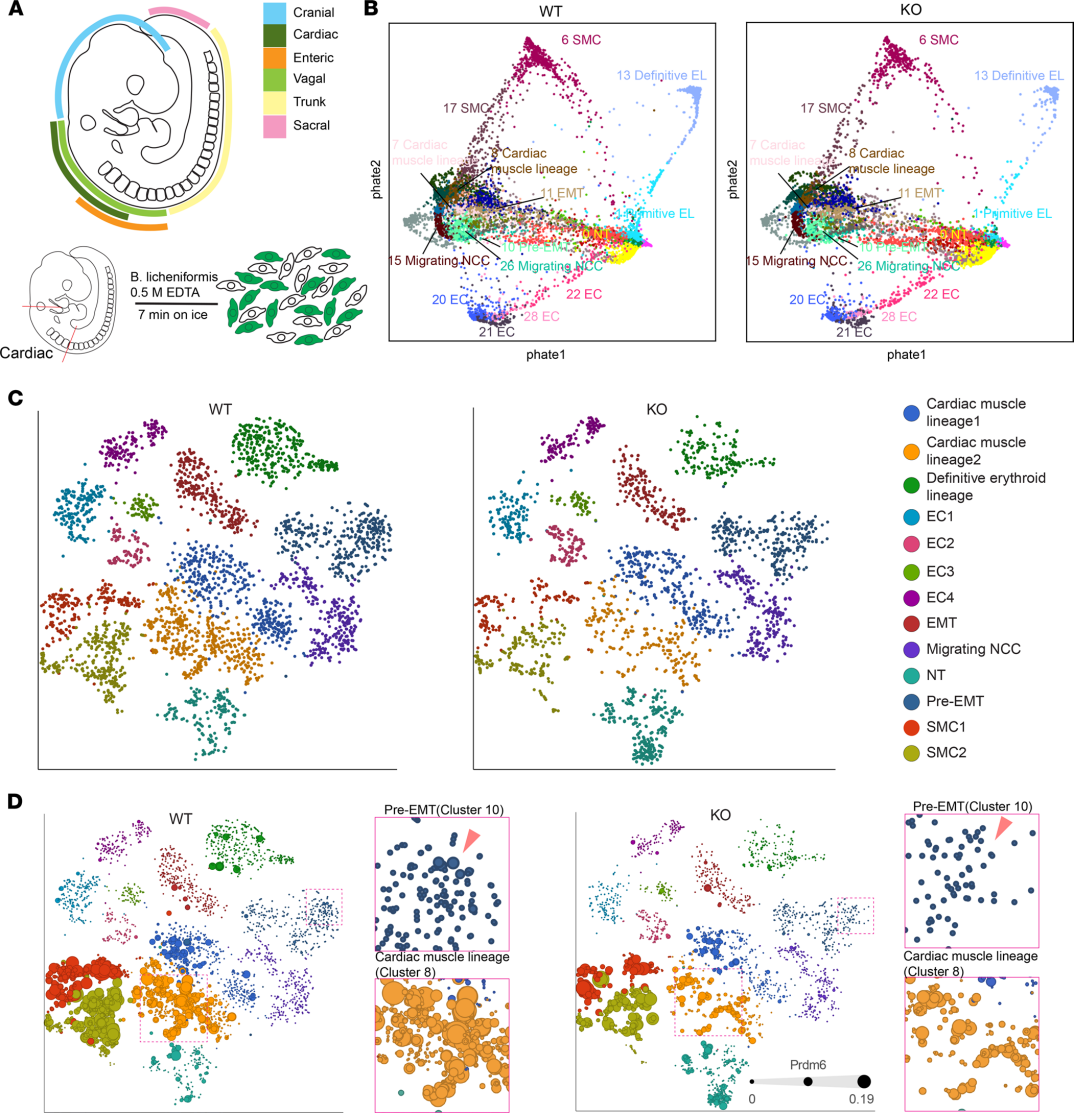
Single-cell RNA-Seq of the cardiac neural crest region. (**A**) Schematic of neural crest segments shown in different colors (top). The segment excised for single-cell RNA-Seq of CNCCs of E9.5 embryo (bottom). (**B**) PHATE embeddings of *Wnt1*-specific KO *Prdm6* mice and the corresponding littermates. (**C**) The t-SNE plots of selected clusters of *Wnt1*-specific KO *Prdm6* mice and the corresponding littermates generated by Partek flow. Each dot represents a cell (https://www.partek.com/partek-flow/). (**D**) The t-SNE plots displaying the expression levels of *Prdm6* in each cluster represented by dot sizes. The insets represent the magnification of pre-EMT (blue); cardiac muscle lineage (yellow) clusters marked by lines (*n* = 3 per group).

The reduced number of ZsGreen1-positive cells in the DA of *Prdm6^fl/fl^ Wnt1-Cre2 ZsGreen1* mice at E17.5 and P0.5 (Figure 3A and Supplemental Figure 2, A and C) suggested impaired migration and/or differentiation of CNCCs. Considering the low levels of Prdm6 transcripts, we embarked on using scRNA-Seq analysis of CNCCs at E9.5 (21). Cardiac NCCs were extracted by dissecting out *Prdm6^fl/fl^ Wnt1-Cre2 ZsGreen1* embryos and their WT littermates (*Prdm6^+/+^ Wnt1-Cre2 ZsGreen1*) from mid otic placode to the posterior edge of the fourth somite (Figure 4A). ScRNA-Seq was carried out using the 10x Genomics Chromium system. The quality control (QC) threshold was made using a modified Seurat pipeline based on 2 QC covariates, count depth and the number of genes per barcode (see Methods). There were an average of 2742 and 3102 counts per cell or an average of 1294 and 1432 unique genes detected in WT and KO conditions, respectively. Only samples with RNA integrity number (RIN) greater than 6.5 were used (see Methods for more details). Graph-based clustering was performed using Seurat, and the PHATE (Potential of Heat-diffusion for Affinity-based Trajectory Embedding) technique developed by Moon and van Dijk et al. (22) was used to visualize trajectory structures. Cluster annotation was made manually based on specific cell markers (Supplemental Table 1).

PHATE-based analyses revealed 31 distinct clusters (Figure 4B and Supplemental Figure 3). There was no obvious difference in the evolution of these clusters from pre–epithelial mesenchymal transition (preEMT)/EMT transition to migrating CNCCs and their terminal differentiation into cardiac and SMC lineages between WT and KO mice clusters. Although the total number of cells was similar between *Prdm6^fl/fl^ Wnt1-Cre2 ZsGreen1* mice and their WT littermates, the number of cells in each cluster varied between the 2 genotypes. Specifically, there were more cells in the neural tube and fewer cells in the EMT and migrating NCC clusters of *Prdm6^fl/fl^ Wnt1-Cre2 ZsGreen1* mice compared with their WT littermates (*Prdm6^+/+^ Wnt1-Cre2 ZsGreen1*) (Figure 4C), consistent with our earlier findings from imaging (Figure 2, B and C). The single-cell transcript analysis showed that *Prdm6* transcript levels were reduced in 2 clusters of the *Prdm6^fl/fl^ Wnt1-Cre2 ZsGreen1* mice compared with WT mice: 1 of 2 cardiac muscle lineages (cluster 8) and pre-EMT (cluster 10) (Figure 4D). Strikingly, in cluster 8, there was reduced expression of SMC markers *Tagln* and *Actg2* and increased expression of endothelial genes and axonal guidance, such as *Sema3c*, *Sema6d, Chd7,* and *CD34* (http://betsholtzlab.org/VascularSingleCells/database.html), in *Prdm6^fl/fl^ Wnt1-Cre2 ZsGreen1* versus WT littermates (Supplemental Figure 4), suggesting impaired differentiation of CNCCs. This was consistent with our earlier observation of ZsGreen1 and VE-cadherin coexpression in the DA of *Prdm6^fl/fl^ Wnt1-Cre2 ZsGreen1* mice at P0.5 (Supplemental Figure 2, A and B). Our findings collectively indicated that loss of *Prdm6* in CNCCs resulted in the presence of fewer differentiated SMCs in the DA due to impaired migration and differentiation of CNCCs.

### Smooth muscle–specific Prdm6-KO mice die postnatally from isolated PDA with altered differentiation of SMCs.

**Figure 5:**
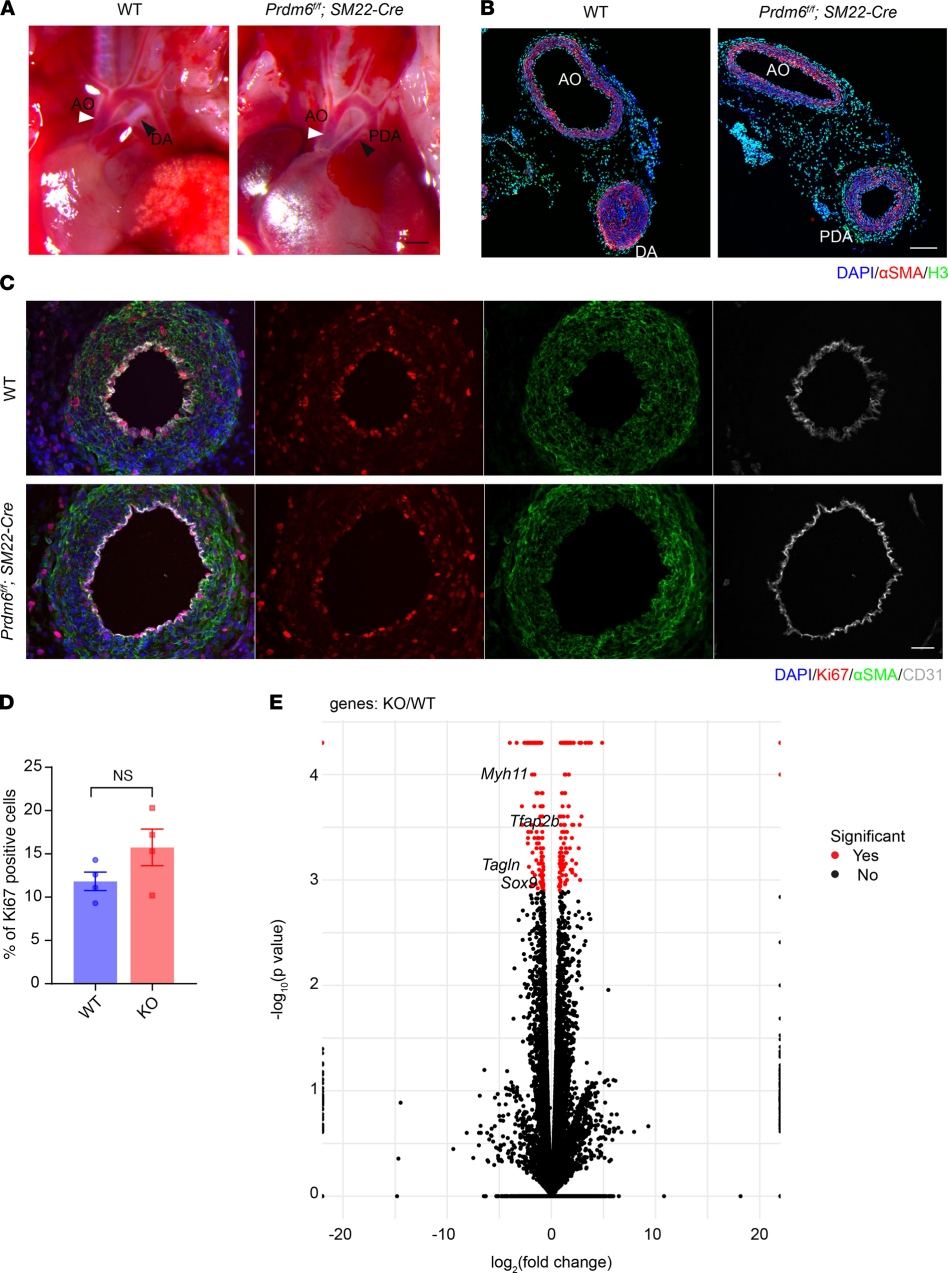
Smooth muscle–specific disruption of *Prdm6* in mice alters transcript levels of contractile proteins and neural crest specifiers and causes patent ductus arteriosus. (**A**) The heart and great cardiac vessels in control (left) and *Prdm6^fl/fl^ SM22-Cre* pups at P0.5 (right); aorta is shown by white arrowheads and the closed DA or patent DA by black arrowheads. Scale bar: 200 μm. (**B**) The confocal images of representative cross-sections of the aorta and DA of control (left) and *Prdm6^fl/fl^ SM22-Cre* (right) P0.5 pups, immunostained for αSMA (red), histone3 (green), and DAPI (blue). Scale bar: 100 μm. (**C**) The confocal images of representative cross-sections of DA of control (top) and *Prdm6^fl/fl^ SM22-Cre* embryos (bottom) at E17.5 immunostained for Ki67 (red) and costained with antibodies against αSMA (green), CD31 (gray), and DAPI (blue). Scale bar: 50 μm. (**D**) Quantification of Ki67-positive cells in the smooth muscle cell layer. (**E**) Volcano plot of bulk RNA-Seq data of *Prdm6^fl/fl^ SM22-Cre* mice DA compared with control mice at E17.5. The log fold change and the log *P* values are shown on *x* and *y* axes, respectively. The comparison between different groups was done by a 2-tailed unpaired *t* test, and data are shown as mean ± SEM. The significantly changed transcripts in *Prdm6^fl/fl^ SM22-Cre* versus control mice are shown in red and the locations of specific genes of interest are shown. The significantly altered transcripts were determined using the following thresholds: adjusted *P* < 0.05, unpaired *t* test, FDR < 0.05, Benjamini-Hochberg corrected. All controls were the corresponding littermates (*n* = 4 per group).

**Figure 6:**
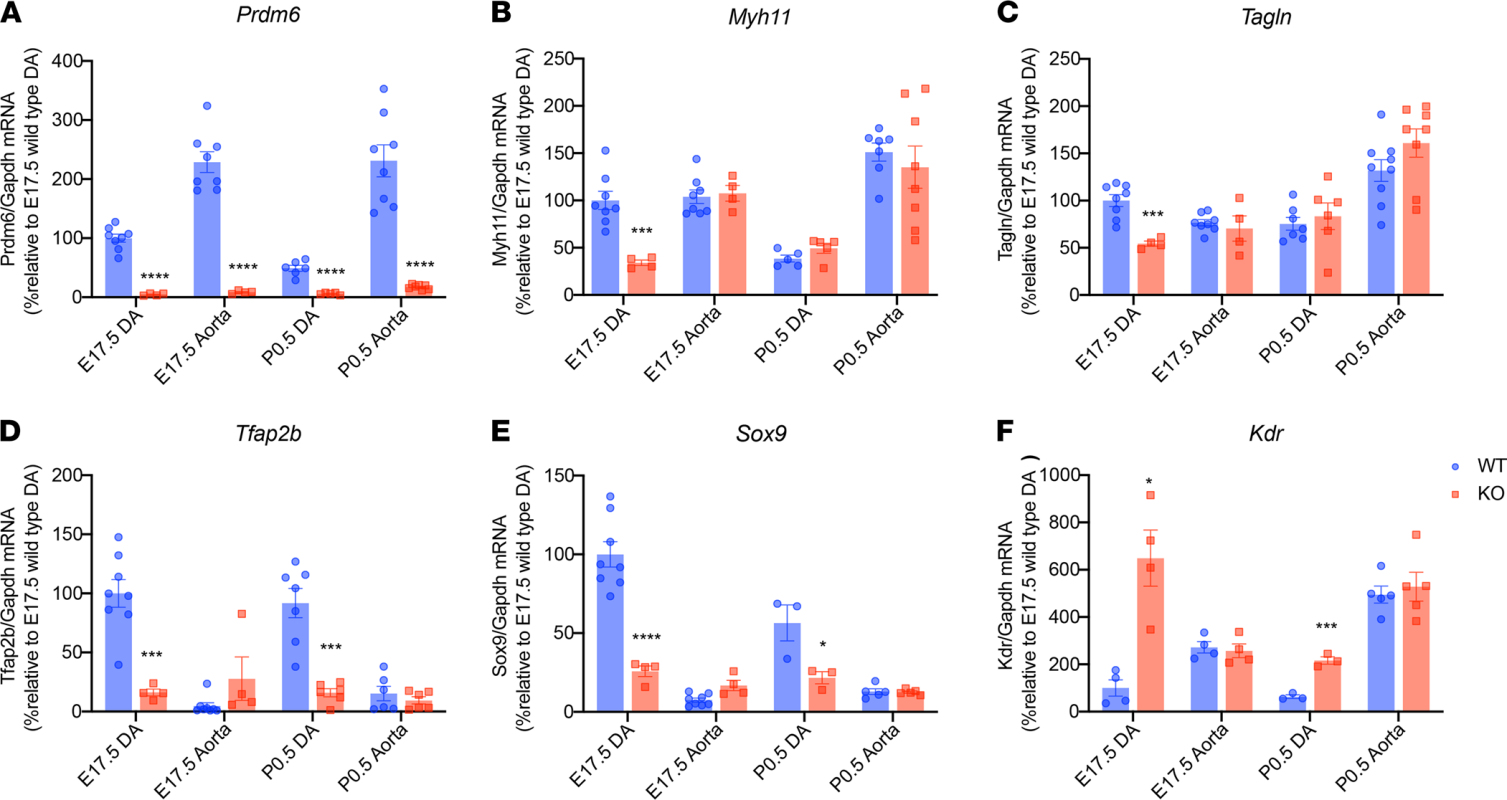
A representation of significantly changed transcripts in *Prdm6^fl/fl^ SM22-Cre* versus control mice measured by qRT-PCR. The relative transcript levels of *Prdm6* (**A**), *Myh11* (**B**), *Tagln* (**C**), *Tfap2b* (**D**), *Sox9* (**E**), and *Kdr* (**F**) in the aorta and DA of *Prdm6^fl/fl^ SM22-Cre* mice versus control mice at E17.5 and P0.5, assayed by real-time fluorescent quantitative PCR are shown. The comparison between different groups was done by a 2-tailed unpaired *t* test, and data are shown as mean ± SEM. Each dot represents a biological replicate. The normalcy was tested by Kolmogorov-Smirnov test. A Mann-Whitney test was conducted for non-normally distributed data. (*n* = 3–9 per group). *P < 0.05, ***P < 0.001, ****P < 0.0001.

To investigate the effect of *Prdm6* disruption on vascular smooth muscle cell (VSMC) differentiation, we crossbred *SM22*-Cre mice with homozygote *Prdm6^fl/fl^* mice to generate *Prdm6^fl/+^ SM22-Cre* mice and then further crossbred to obtain *Prdm6^fl/fl^ SM22-Cre* mice. Homozygous *Prdm6^fl/fl^ SM22-Cre* mice showed no obvious growth retardation or syndromic features (Supplemental Figure 5), but all died at P1.5, slightly later compared with *Prdm6^fl/fl^ Wnt1-Cre2 ZsGreen1* mice. Gross anatomical examination of the cardiovascular system showed a PDA, albeit with a smaller diameter compared with *Prdm6^fl/fl^ Wnt1-Cre2 ZsGreen1* mice (Figure 5A). The cross-section examination of the DA showed complete patency of DA (Figure 5B), a completely penetrant trait in both male and female mice (Table 2). We assessed cell proliferation in the DA at E17.5 using Ki67, when ductal cells were still viable in WT mice. Once again, we detected no significant difference in Ki67 staining between *Prdm6^fl/fl^ SM22-Cre* and WT littermates (Figure 5, C and D). This was a striking finding given the patency phenotype and suggested that the loss of *Prdm6* in VSMCs impairs their differentiation but not proliferation. Consistent with this, bulk RNA-Seq analysis of E17.5 DA from *Prdm6^fl/fl^ SM22-Cre* mice and WT littermates identified contractile proteins, such as *Tagln* and *Myh11,* as the most downregulated genes in the DA of *Prdm6^fl/fl^ SM22-Cre* mice versus WT littermates (Figure 5E and Supplemental Figure 6) from 280 differentially expressed genes (Supplemental Table 2). Strikingly, the expression levels of *Tfap2b* and *Sox9*, which also function as NCC specifiers, were significantly reduced in the DA of *Prdm6^fl/fl^ SM22-Cre* versus WT littermates (Figure 5E and Supplemental Figure 6). Real-time quantitative PCR (qRT-PCR) further confirmed the reduced expression of *Prdm6* (Figure 6A) in both the DA and aorta. However, the transcript levels of contractile proteins *Myh11* and *Tagln* were reduced in the DA and not the descending aorta of *Prdm6^fl/fl^ SM22-Cre* mice at E17.5 (Figure 6, B and C). The expression levels of *Tfap2b* and *Sox9* were also decreased, whereas those of the endothelial marker *Kdr* (also known as *Flk1*, *Vegfr2*) were increased at E17.5 and P0.5 in the DA (Figure 6, D–F). These findings further support the role of *Prdm6* as a regulator of SMC differentiation during cardiovascular development. Although *Prdm6^fl/fl^ SM22-Cre* mice had smaller DA size and lumen compared with *Prdm6^fl/fl^ Wnt1-Cre2 ZsGreen1* mice, they died from PDA, signifying the importance of SMC differentiation in DA closure.

To identify the *Prdm6-*regulated gene network, we applied gene set enrichment analysis by using Gene Ontology (GO). Two separate analyses using genes significantly upregulated and downregulated were carried out. Vasculature development, blood vessel development, smooth muscle tissue development, collagen fibril organization, extracellular matrix assembly, and cell-matrix adhesion were the most significantly downregulated, and cell-cell adhesion and negative regulation of cell differentiation were among the most significantly upregulated pathways (Supplemental Table 3). We assessed the protein levels of selected differentially expressed genes by immunofluorescent staining of the DA of *Prdm6^fl/fl^ SM22-Cre* versus WT littermates at E17.5. We found that the SMC contractile protein Myh11 and fibronectin, a protein synthesized and secreted from SMCs, were significantly reduced in the DA of *Prdm6^fl/fl^ SM22-Cre* versus WT littermates (Supplemental Figure 7, A and B). Fibronectin has been shown to be synthesized to a significantly greater degree in the DA compared with the aorta at early gestation and contributes to the enhanced migration of DA SMCs (23).

### Impaired CNCC migration in Prdm6^fl/fl^ mice results from deregulated Wnt1 signaling.

**Figure 7:**
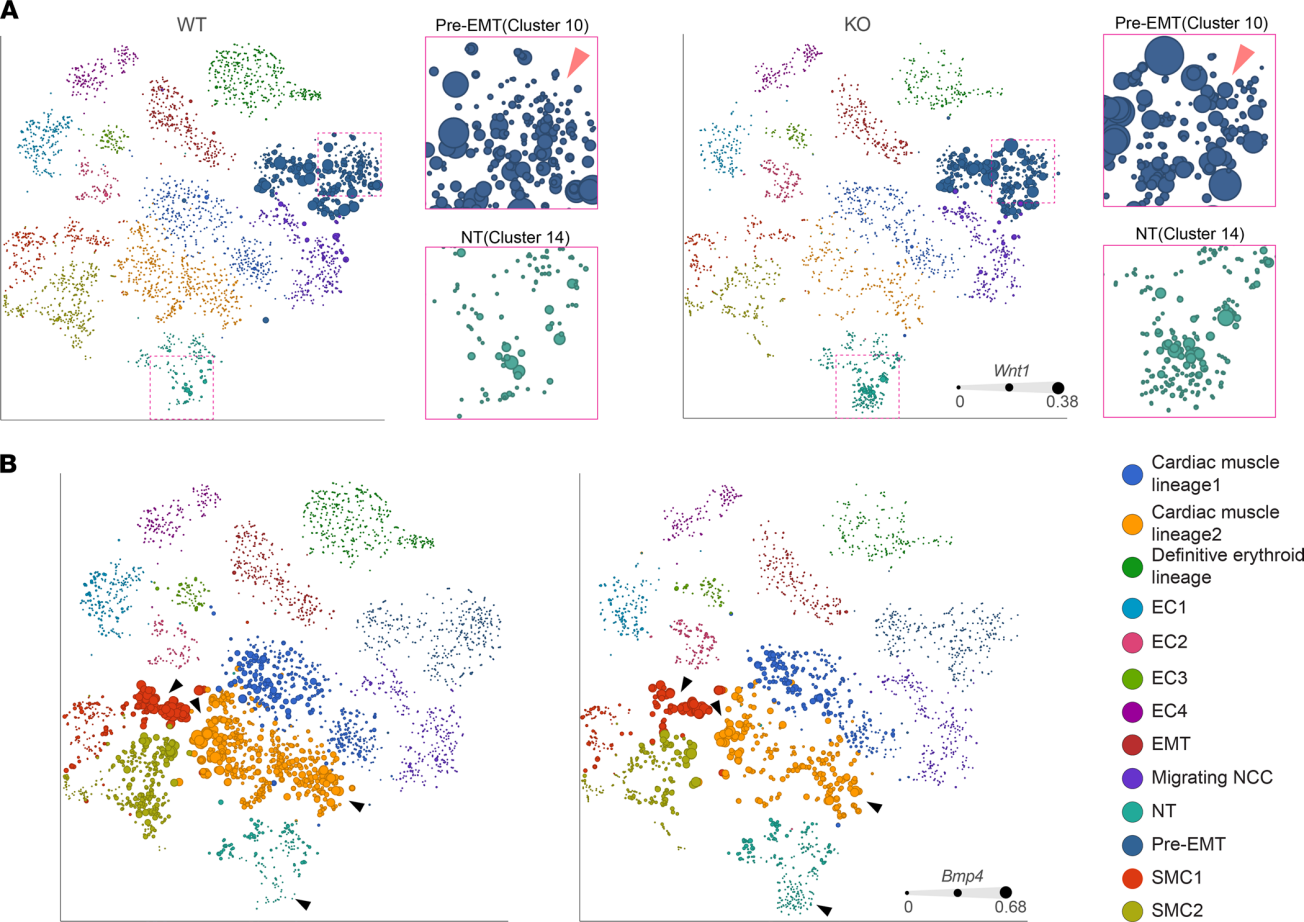
Single-cell RNA-Seq of the cardiac neural crest region of the embryo. (**A**) The t-SNE plots displaying the expression levels of *Wnt1* in each cluster represented by dot sizes. The insets represent the magnification of pre-EMT (blue) and neural tube (green) clusters. (**B**) The t-SNE plots highlighting the reduced expression levels of *Bmp4* in neural tube and cardiac muscle lineages of *Wnt1-*specific KO *Prdm6* compared with the corresponding littermates. Expression levels of *Bmp4* represented by dot sizes.

The induction and subsequent specification of NCCs involves the combinatorial input of multiple signaling pathways. WNT and BMP signaling are key inducers of the neural crest, but their role in CNCC fate and migration has not been well studied. We found that expression levels of *Wnt1* were highest in the pre-EMT cluster (cluster 10), followed by 1 of the 4 neural tube clusters (cluster 14), and dramatically reduced after NCC delamination from the neural tube (Supplemental Figure 8A). *Wnt1* transcript levels were higher in *Prdm6^fl/fl^ Wnt1-Cre2 ZsGreen1* versus WT littermates (*Wnt1-Cre2 ZsGreen1*) in several clusters, including the pre-EMT cluster (Figure 7A), which suggested that *Prdm6* may function to suppress *Wnt1* during emergence of CNCCs. Pathway analysis of the pre-EMT cluster showed the “Development positive regulation of Wnt/β-catenin signaling at the receptor level” as one of the most significantly upregulated pathways (Supplemental Table 4).

*Bmp* signaling has been implicated in maintaining NCC identity by counteracting the effect of Wnt signaling (24). Accordingly, we found reduced levels of *Bmp4* in the neural tube, cardiac muscle lineages, and SMC clusters upon loss of *Prdm6* compared with littermate controls (Figure 7B, black arrowheads; Supplemental Figure 8B), concomitant with increased *Wnt1* levels. This indicated that *Prdm6* controlled a balance between BMP/Wnt signaling, which was impaired in CNCCs of *Prdm6^fl/fl^* mice and impeded their delamination and migration.

**Figure 8:**
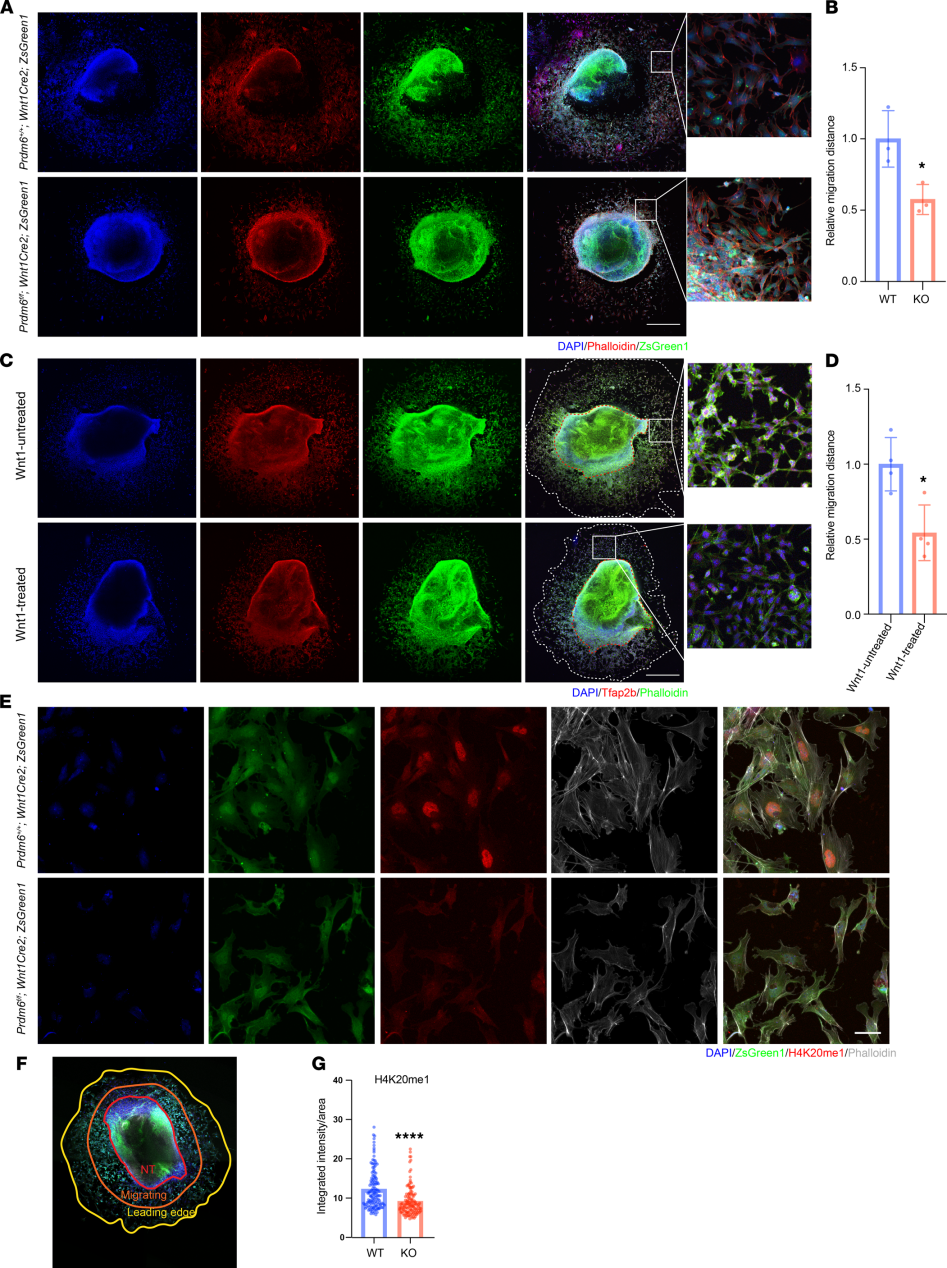
Ex vivo examination of neural crest migration. (**A**) Migration of NCCs from ex vivo cultured neural tube of WT (*Prdm6^+/+^ Wnt1-Cre2 ZsGreen1*) and KO (*Prdm6^fl/fl^ Wnt1-Cre2 ZsGreen1*) mice at E9.5. The NCCs are shown as ZsGreen1-positive (green) and the neural tube is stained with DAPI (blue) and phalloidin (red). Scale bar: 500 μm. (**B**) Quantification of migration distances; each dot represents a biological replicate. Migration is measured as distance from the periphery at the junction with 2 perpendicular radiuses. (**C**) Migration of NCCs from ex vivo cultured neural tube of WT mice at E9.5 treated with WNT1 for 24 hours and stained with DAPI (blue), Tfap2b antibody (red), phalloidin (green). Scale bars: 500 μm. (**D**) Quantification data of **C**. Each dot represents a biological replicate. (**E**) H4K20 monomethylation (red) of NCCs in the leading edge of ex vivo cultured neural tube of WT (*Prdm6^+/+^ Wnt1-Cre2 ZsGreen1*) and KO (*Prdm6^fl/fl^ Wnt1-Cre2 ZsGreen1*) mice at E9.5 stained with DAPI (blue) and phalloidin (gray). Scale bar: 50 μm. (**F**) Schematic depiction of ZsGreen1-positive (green) migrating NCCs, divided into 2 populations of migrating (clustered) and leading edge (single cells). (**G**) Quantification of H4K20 monomethylation from **E**; each dot represents a cell. The comparison between different groups was done by a 2-tailed unpaired *t* test, and data are shown as mean ± SEM. *P < 0.05*,* ****P < 0.0001. All controls were the corresponding littermates (*n* = 3 per group).

To establish the role of Prdm6 in NCC migration, we cultured excised neural tubes of the E9.5 WT and *Prdm6^fl/fl^ Wnt1-Cre2 ZsGreen1* embryos ex vivo to assess migration of CNCCs. We observed a marked reduction of NCC migration after 24 hours in *Prdm6^fl/fl^ Wnt1-Cre2 ZsGreen1* versus WT littermates (Figure 8, A and B). Postulating that the increased *Wnt1* expression underlies the CNCC migration defect, we examined whether the treatment of WT neural tube tissue ex vivo with Wnt1 could phenocopy the Prdm6*^fl/fl^* condition. We therefore repeated these experiments using WT neural tubes and treated them with WNT1(100 ng/mL) for 24 hours. In response, we observed reduced migration of the delaminated NCCs compared with the PBS control, supporting our hypothesis (Figure 8, C and D). These findings are consistent with a prior report that constitutive activation of β-catenin in the NCC-derived SMC precursor results in a reduced NCC-derived population of SMCs in the DA (20), leading to its patency. Our findings also identified excess Wnt activation as a mechanism for PDA.

### Loss of Prdm6 impairs H4K20 methylation and G1-S phase progression.

We next focused on the molecular mechanisms that could link loss of *Prdm6* to altered gene expression. We and others had previously shown that PRDM6 regulates H4K20 methylation (9, 25). We compared H4K20 monomethylation of *Prdm6^fl/fl^ Wnt1-Cre2 ZsGreen1* mice CNCCs at E9.5 with those of the WT littermates ex vivo, and consistent with earlier findings, we observed reduced H4K20 monomethylation in the solitary leading edge of *Prdm6^fl/fl^ Wnt1-Cre2 ZsGreen1* CNCCs (Figure 8, E–G). There was no significant difference of H4K20 monomethylation in early migrating NCCs (Supplemental Figure 9, A and B). Together, these results suggest that loss of *Prdm6* reduced H4K20 methylation in CNCCs.

**Figure 9:**
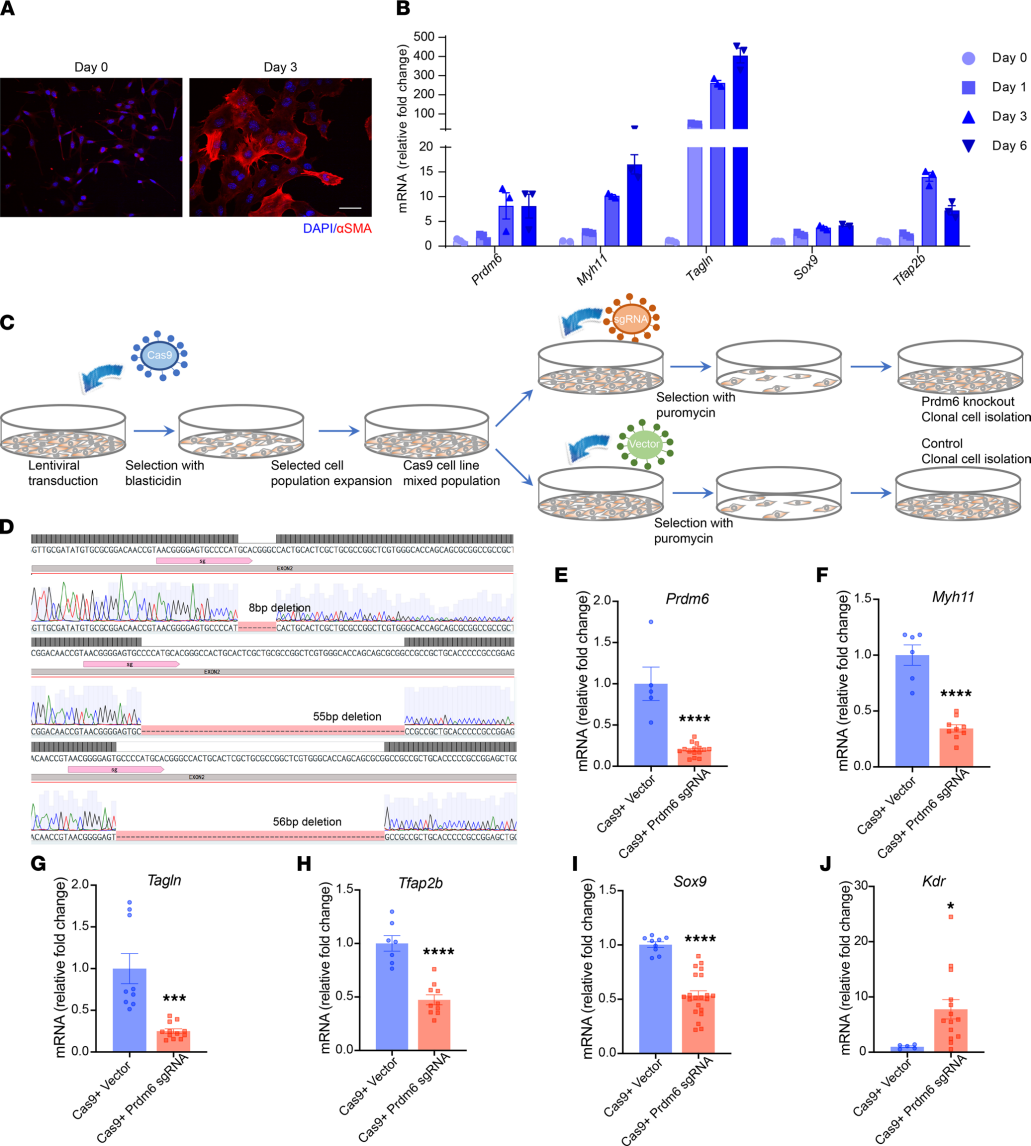
Characterization of *Prdm6* in mouse NCC line. (**A**) Confocal images of O9-1 NCC line differentiated into smooth muscle cells after transfer to chemically defined medium at day 0 and day 3 and stained for αSMA (red) and DAPI (blue); scale bar: 50 μm. (**B**) mRNA level of specified genes in O9-1 cells at days 0, 1, 3, and 6 of differentiation relative to *Gapdh*, quantified by qRT-PCR. (**C**) Schematic of screening of O9-1 transduced with Cas9 lentiviral vectors. Cell expressing Cas9 were selected with blasticidin, transduced with sgRNA lentiviral vectors, and selected with puromycin to establish clonal cell lines. (**D**) Images of Sanger sequencing of segments of *Prdm6* genes from 3 clonal cell lines with 8bp, 55bp, or 56bp deletions. All 3 clonal cell lines were used in the experiment. qRT-PCR measurements of *Prmd6* (**E**), *Myh11* (**F**), *Tagln* (**G**), *Tfap2b* (**H**), *Sox9* (**I**), and *Kdr* (**J**) mRNA expression in control and Cas9 + Prdm6 sgRNA cell lines. Each dot represents a biological replicate. Data are mean ± SEM. For statistical analysis, groups were compared using unpaired 2-tailed *t* test. *P < 0.05, ***P < 0.001, ****P < 0.0001.

**Figure 10:**
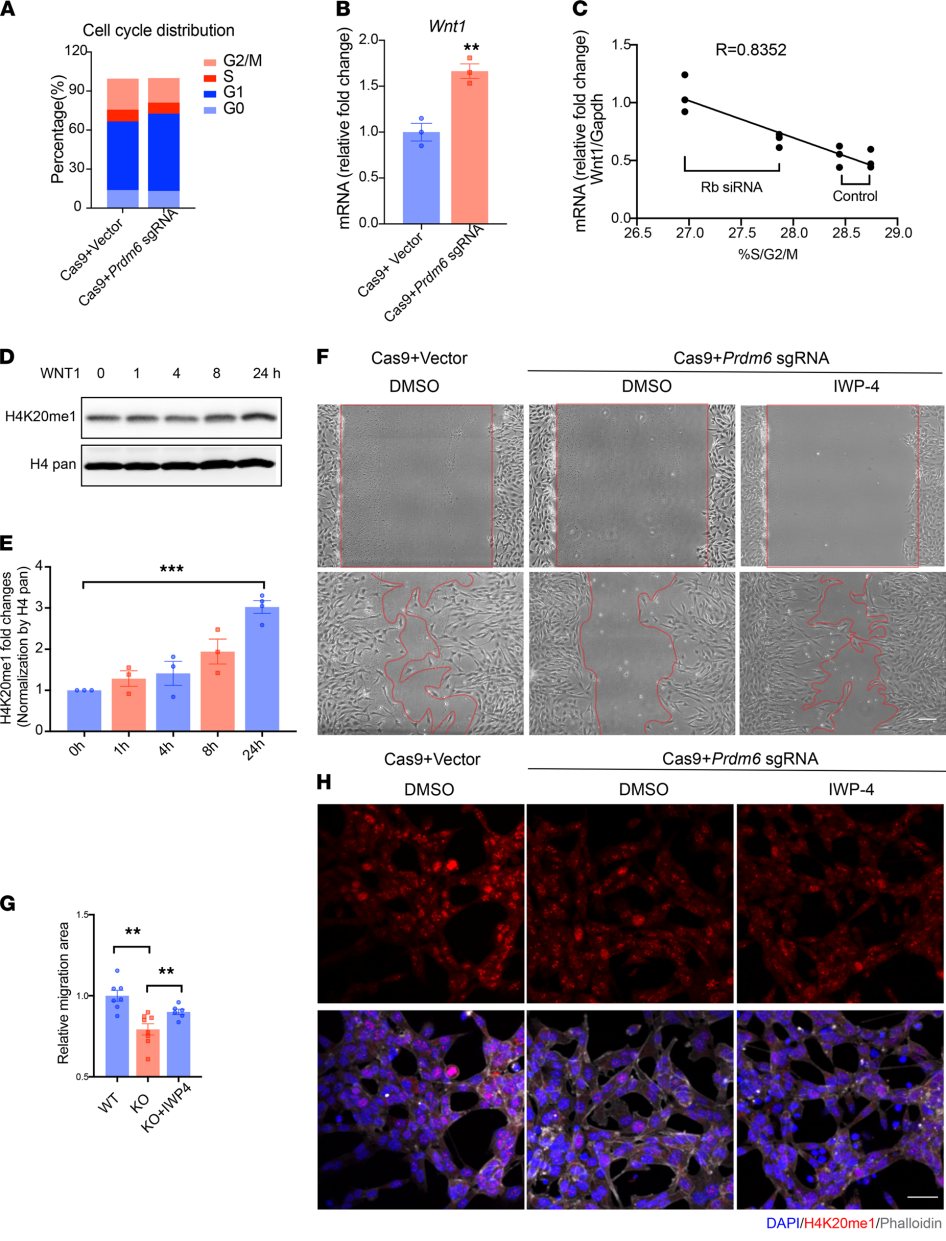
The association between loss of *Prdm6*, elevated *Wnt1*, and reduced H4K20me1 in a mouse NCC line. (**A**) Images of light scattering by flow cytometry FACS demonstrating the cell cycle of control and *Prdm6*-KO O9-1 cells. (**B**) *Wnt1* mRNA expression levels measured by qRT-PCR in WT and *Prdm6* CRISPR KO O9-1 cells. (**C**) The mRNA expression levels of *Wnt1* and its relationship to percentage of cells in S/G2/M phase in O9-1 cells either treated with *Rb*-siRNA or control siRNA. (**D** and **E**) H4K20me1 level of cells treated with PBS and WNT1 assayed by Western blot and its quantification (**E**). (**F**) Migration of *Prdm6*-deficient O9-1 cells in response to Wnt inhibitor IWP-4 compared with DMSO by wound assay and their quantification (**G**). Scale bar: 50 μm. (**H**) The confocal image of H4K20me1 in *Prdm6*-deficient O9-1 cells treated with Wnt inhibitor (IWP-4) or DMSO by staining DAPI (blue), phalloidin (gray), and antibodies against H4K20me1 (red). Scale bar: 50 μm. Each dot represents a biological replicate. The comparison between different groups was done by a 2-tailed unpaired *t* test, and data are shown as mean ± SEM. The comparisons between multiple groups were done by 1-way ANOVA. A Mann-Whitney test was conducted for non-normally distributed data (*n* = 3–8 per group). **P < 0.001, ****P < 0.0001.

Interestingly, H4K20 monomethylation (H4K20me1) has been shown to promote G1 to S cell cycle progression (26), which is an essential step for the delamination of premigratory NCCs (27). H4K20me1 at E- and N-cadherin promoters has also been shown to regulate their transcription and mediate EMT (28). We therefore hypothesized that H4K20me1 by *Prdm6* promotes cell cycle progression and alters the expression of a gene network that is necessary for delamination and migration of the CNCCs. To test this, we used CRISPR/Cas9-based gene editing to inactivate *Prdm6* in exon2 of O9 mouse NCCs in vitro. We first differentiated these cells into SMCs (Figure 9A) and verified their competency in expressing *Prdm6*, contractile proteins *Myh11* and *Tagln*, and NCC specifiers *Sox9* and *Tfap2b* at days 0, 1, 3, and 6 (Figure 9B). Subsequently, we infected the cells with lentivirus containing CRISPR/Cas9 and transfected them with either control vector or 3 different sgRNAs targeting *Prdm6* exon2 (Figure 9C) and generated 3 clonal cell lines with 8, 55, and 56 bp deletions (Figure 9D). qRT-PCR expression analysis confirmed the loss of *Prdm6* expression and subsequent downregulation of contractile proteins *Myh11* and *Tagln* and NCC specifiers *Tfap2b* and *Sox9,* as well as upregulation of the endothelial maker *Kdr* in all *Prdm6*-deficient lines (Figure 9, E–J), consistent with our in vivo gene expression analyses. We then subjected the *Prdm6*-deficient CNCCs to FACS analysis to assess the cell cycle activity and observed a marked reduction in G1-S cell cycle progression compared with WT O9 cells (Figure 10A). Our analyses revealed a greater number of *Prdm6*-deficient CNCCs in the G1 phase and fewer in the S and G2/M phase compared with WT CNCCs, indicating G1-S phase arrest. This finding was associated with increased *Wnt1* expression in *Prdm6*-KO versus WT O9 cells (Figure 10B).

To determine the direct role of H4K20me1 in regulating CNCC cell cycle progression, we inactivated retinoblastoma-associated protein Rb1 in O9 cells using Rb1-specific siRNA. RB1 protein plays a critical role in recruiting H4K20 methyltransferases, such as Suv4-20h1/KMT5B and Suv4-20h12/ KMT5C29, and mediates mono-, di-, and tri-methylation of H4K20 (29). Knockdown of Rb1 in O9 cells resulted in modest but persistently reduced G1-S progression compared with control siRNA, as determined by FACS analysis (Figure 10C). There was an inverse relationship between G1-S progression and *Wnt1* expression (R = 0.83; Figure 10C).

Our data suggested that loss of *Prdm6* reduced H4K20me1 and increased *Wnt1* levels, impeding CNCC migration, but the order of the events remained unknown. We examined the effect of Wnt1 on histone modifications in these cells and found that it increased H4K20me1 levels compared with PBS, as predicted (Figure 10, D and E). This finding suggests that reduced H4K20me1 after *Prdm6* knockdown is not caused by increased Wnt1. Accordingly, treatment of *Prdm6*-deficient O9 cells with Wnt inhibitor (IWP-4, 5 μM, 04-0036) rescued the migration-defective phenotype (Figure 10, F and G); however, it did not change H4K20me1 (Figure 10H). In conclusion, we suggest a model whereby Prdm6-H4K20me1 synchronizes the cell cycle, which is necessary for the regulation of Wnt1 transcript levels and CNCC migration. In contrast, the loss of Prdm6 results in reduced H4K20 monomethylation and G1-S cell cycle progression, resulting in increased Wnt1 expression and impaired CNCC migration.

## Discussion

NCCs give rise to a diverse cell lineage and therefore are increasingly gaining attention for their potential role in tissue regeneration. The mouse phenotyping and fate mapping in our study showed that *Prdm6* regulates differentiation and migration of CNCCs, which are the precursor of SMCs in the tunica media of the DA and pharyngeal arches (16). Consequently, loss of Prdm6 results in early postnatal death from PDA (30–32). The importance of CNCCs in DA closure has been previously shown by the ablation of myocardia in early postmigratory NCC derivatives, albeit the exact molecular mechanisms remain to be elaborated (32). Mutations in histone modifiers *KMT2D* (*histone-lysine N-methyltransferase 2D*) and *KDM6A* (*lysine-specific demethylase 6A*) (33) and *EHMT1* (euchromatic histone*-lysine N-methyltransferase 1*) (34) have also been associated with syndromic congenital heart disease due to defects in NCC development (35). However, in contrast to *PRDM6* mutations, defects in these genes appear to affect both cardiac and cranial NCCs.

Our investigations ex vivo demonstrated that increased *Wnt1* underlies impaired CNCC migration in *Prdm6*-deficient mice, which was rescued by Wnt inhibition. These findings are consistent with a prior report that constitutive activation of β-catenin (ctnnb1Δex3) results in a reduced NCC-derived population of SMCs in the DA and early postnatal death (20). Although NCC migration in ctnnb1Δex3 was not explored, our fate mapping and functional studies suggest that excessive Wnt activation can result in impaired CNCC migration.

Our comprehensive genomic and epigenomic approach identified a Prdm6-dependent gene regulatory network that underlies the regulation of a subset of CNCCs during development. The neural crest gene regulatory networks consist of multiple complex subnetworks that are sequentially activated to control the key steps of neural crest formation, delamination, migration, and differentiation. Of particular importance is the mutually inhibitory crosstalk between Wnt and Bmp signaling in that Bmp counteracts proliferation promoted by Wnt while Wnt antagonizes BMP-dependent neuronal differentiation (36). The coordinated actions of these 2 pathways result in activation of transcriptional cascades that drive EMT and migration and cooperate with signaling systems that induce cell differentiation (37). In our models, we showed that the absence of *Prdm6* tips the balance in favor of Wnt1 by reducing *Bmp4* and increasing *Wnt1* transcripts in several clusters, leading to impaired CNCC migration.

*PRDM6* has been previously shown by our group and others to mediate H4K20 methylation in vitro (9, 25). H4K20 monomethylation has been shown to be critical for cell cycle progression (38, 39). In the current study, we showed that Prdm6 regulates H4K20 methylation in migrating NCCs and that this event is critical for cell cycle progression and subsequent induction of EMT and migration of specific CNCCs. We showed that H4K20 methylation and cell cycle activation are inversely related to *Wnt1* expression. The causal relationship between H4K20 methylation, cell cycle activation, and *Wnt1* expression was supported by knocking down the retinoblastoma-associated protein Rb1, which has been shown to interact with H4-K20 methyltransferases Suv4-20h1/KMT5B and Suv4-20h12/ KMT5C (40) and to mediate their methylation of H4K20 (41). Accordingly, the knockdown of Rb1 led to G1-S phase arrest and was associated with higher *Wnt1* expression. These findings linked reduced H4K20 methylation in *Wnt1-Cre2 Prdm6^fl/fl^* mice to increased *Wnt1* expression in multiple CNCC clusters.

One of the most important implications of our study is the role of temporal and spatial epigenetic regulation in the specification of NCCs. It is likely in this context that elevated Wnt1 impairs a subset of CNCCs that contributes to the DA, whereas elevated Wnt1 is a necessary component of trunk NCCs, including their entry into the S-phase and their facilitated migration (42). Strikingly, environmental factors, such as teratogens and infectious disorders during the first trimester of pregnancy, have been associated with isolated persistent PDA and altered DNA or histone modifications (43–46). The characterization of Prdm6 function in NCCs has, therefore, a broad implication for the pathogenesis of inherited and acquired congenital heart diseases and may lead to identification of novel targets for drug development.

## Methods

### Generation of SM22-Cre KO mice.

The day of plug detection was considered as E0.5. *Prdm6*-*flox* mice were generated in-house and were intercrossed to generate homozygous *Prdm6*-*flox* mice (*Prdm6^fl/fl^*). *Prdm6^fl/fl^* mice were viable, fertile, and indistinguishable from control littermates. *Prdm6^fl/fl^* mice were intercrossed with *SM22-Cre* transgenic mice that expressed high levels of Cre-recombinase in SMCs (Supplemental Figure 10).

### Neural crest–restricted inactivation of the Prdm6 gene.

To determine the function of *Prdm6* in cardiac neural crest–derived cells, homozygous *Prdm6^fl/fl^* mice were crossed with transgenic mice expressing Cre-recombinase under the transcriptional control of the neural crest–restricted *Wnt1* promoter. *Prdm6^fl/+^ Wnt1-Cre2* offspring were then interbred with *Prdm6^fl/+^* mice to generate *Prdm6^fl/fl^ Wnt1-Cre2* mutants and control littermates *Prdm6^+/+^ Wnt1-Cre2* (Supplemental Figure 11).

For lineage tracing, *Prdm6^fl/fl^* mice were crossed with ZsGreen1 to generate *Prdm6^fl/+^ ZsGreen1* mice and then interbred with *ZsGreen1* to generate *Prdm6^fl/+^ ZsGreen1/ZsGreen1* mice. To knock out *Prdm6* in *Wnt1*-positive NCCs, the *Prdm6^fl/+^ ZsGreen1/ZsGreen1* mice were intercrossed with *Prdm6^fl/+^ Wnt1-Cre2* mice (Supplemental Figure 12).

*Wnt1-Cre2* mice were purchased from The Jackson Laboratory, stock number 022501 (full strain name *B6.Cg-E2f1Tg(Wnt1-cre)2Sor/J*). *ZsGreen1* mice were received from Daniel Greif (Yale University) and are also available from The Jackson Laboratory (*Rosa-CAG-LSL-ZsGreen1-WPRE*, Jax 007906). All mice were fed ad libitum and housed at constant ambient temperature in a 12-hour light/12-hour dark cycle. Both male and female mice were studied. All studies in animals were conducted in accordance with the NIH *Guide for the Care and Use of Laboratory Animals* (National Academies Press, 2011).

### Mouse embryo and tissue preparation and IHC.

Mouse embryos and tissues were collected and rinsed in ice-cold Dulbecco’s PBS and fixed by incubation for 15–20 minutes in PBS containing 4% PFA (Santa Cruz, sc-281692) at 4°C. They were rinsed twice in ice-cold PBS and incubated for 24 hours in 30% sucrose/PBS at 4°C, followed by Tissue-Tek OCT compound (catalog 4583) in plastic molds and allowed to freeze on dry-ice and stored at –80°C. The tissue submerged in OCT compound was sectioned onto 5 μm–10 μm slices in a cryostat and dried on Fisherbrand Superfrost Plus slides (Fisher Scientific, catalog 22-037-246) 12 to 16 hours at room temperature. P0.5 mice tissue were collected after anesthesia. Hearts were then removed and treated as described above.

### Immunofluorescence.

All immunofluorescence staining was performed on 5 μm–10 μm frozen sections. For immunostaining, the slides were washed 3 times in 1× PBS, followed by 3 washes in PBS plus 0.1% Triton X-100 and incubated in the blocking solution containing 5% BSA plus 1× PBS plus 0.1% Triton X-100 for 2–3 hours. Subsequently, the slides were incubated in the primary antibody overnight, followed by 3 washes in PBS plus 0.1% Triton X-100 for 10 minutes each. After 2 hours of secondary antibody incubation, slides were washed 3 times in PBS plus 0.1% Triton X-100 for 10 minutes each. The specimens were overlaid with ProLong-Gold Antifade solution and a coverslip. All primary antibodies (Supplemental Table 5) were diluted 1:300 and secondary antibodies at 1:300. As a control, we used no primary as well as PRDM6 KO mice tissues. Otherwise, only validated and previously published antibodies were used. Fluorescence images were obtained by a Zeiss 4 laser confocal microscope or Leica SP8 confocal microscope, and the intensity was measured using the same laser output, gain, and offset for each set of antibodies tested. Images were quantified with ImageJ (NIH) and adjusted for the area.

### Whole-mount staining.

Whole-mount microscopy was carried out as previously described (47). Briefly, E9.5 mice were harvested and kept in ice-cold PBS for 5 minutes to drain blood from the umbilical cord and then fixed in 4% PFA at 4°C and washed at room temperature. Samples were dehydrated in methanol/PBS series (20%, 40%, 60%, 80%) for 30 minutes at room temperature and then 100% for the entire day. Samples were chilled over ice and bleached in 5% H_2_O_2_ in methanol overnight at 4°C, washed with 80% methanol/PBS for 30 minutes at room temperature, and rehydrated with the methanol series in PBS/0.2% Triton X-100 (fresh prepared): 80%, 60%, 40%, 20%, 0%; 30 minutes each at room temperature. For immunolabeling, pretreated samples were incubated in 1× PBS/0.2% Triton X-100/20% DMSO/0.3 M glycine at 37°C for 24 hours and blocked in 1× PBS/0.2% Triton X-100/10% DMSO/6% goat serum at 37°C for 24 hours and subsequently washed with 1× PBS/0.2% Tween 20 with 10 μg/mL heparin and incubated with primary antibody in 1× PBS/0.2% Tween 20 with 10 μg/mL heparin/5% DMSO/3% donkey serum at 37°C for 24 hours. After several washes, samples were incubated with secondary antibody in 1× PBS/0.2% Tween 20 with 10 μg/mL heparin/3% donkey serum at 37°C for 24 hours. For clearing, samples were incubated overnight in 50%, 80%, and 100% tetrahydrofuran/H_2_O (THF, Sigma-Aldrich, 186562-12X100ML) for 2 hours each time. Samples were then incubated in dichloromethane (DCM, Sigma-Aldrich, 270997-12X100ML) until they sunk to the bottom for 5 minutes and in dibenzyl ether (Sigma-Aldrich, 108014-1KG) until the samples were clear (20 minutes to 2 hours). We then examined samples using light sheet microscopy.

### Isolation and culture of the neural tube.

Isolation of the neural tube was carried out as described by Elise R. Pfaltzgraff, Nathan A. Mundell, and Patricia A. Labosky (48). For timed pregnancies, dams were checked in the morning for a vaginal plug and the embryo was considered as E0.5 at noon of the day the plug was observed. Mice were euthanized, and the embryos were removed at E9.5 and placed on ice-cold sterile DPBS. Using insulin needles, neural tubes were cut between the mid otic placode and the posterior edge of the fourth somite. The yolk sacs were kept for genotyping and the remaining embryonic tissues were discarded. The cardiac neural tube–containing segments were placed in collagenase/Dispase (Sigma-Aldrich, catalog 269-638) at room temperature for 8 minutes and subsequently washed in ice-cold sterile DPBS. The non-neural ectoderm was gently removed from the tissue, and the somites were separated away from the cardiac neural tube. The remaining parts of the mesoderm were gently removed using a cut-down 1 mL pipette tip. The isolated neural tube was washed for 30 seconds with culture medium (ES101-B) with fibroblast growth factor (GF003) and placed in the center of Matrigel-coated (Corning, catalog 354234) 24-well glass-bottom cell culture plate (Nest Scientific, catalog 801006) and incubated at 37°C.

### RNA extraction, cDNA synthesis, and qPCR.

The RNA was extracted using Qiagen miRNeasy Micro kit (catalog 1071023). Next, 100–500 ng of RNA was used for cDNA synthesis (iScript cDNA synthesis, Bio-Rad, 1708890). No-RT and no-RNA controls were used to ensure DNase digestion and no residual contamination. The cDNA was diluted to different concentrations and eventually were diluted by 5-fold to obtain qPCR values in the linear range. qPCR primers (Supplemental Table 6) were from PrimerBank. A dilution curve that included primers and qPCR Master Mix were utilized to ensure that PCR primers did not self-anneal and to reduce background amplification. The qPCR was performed with 2× SYBR Green Master Mix (Bio-Rad, 170-8880), 0.5 μM primer (forward and reverse), and 5 μL of 1:5 diluted cDNA and rest water. All qPCR reactions had 3 technical replicates and at least 3 biological replicates per genotype. All values were normalized to the reference gene *Gapdh*.

### Bulk RNA-Seq.

The reads were trimmed for quality and length. All the reads were minimum base quality of Q30, and reads with minimum length of 45 were used for further analysis. The reads were aligned to the mouse UCSC reference genome mm10 (49, 50) using TopHat2 (51). The alignment data were converted to per-gene counts using Cufflinks (52) and further analyzed using Cuffdiff and R (http://www.r-project.org/index.html). The results were visualized using R.

### ScRNA-Seq.

E9.5 embryos were harvested and briefly placed in ice-cold DPBS and then placed on a plastic petri dish resting on ice. Using insulin needles, the cardiac neural crest was dissected out from the mid otic placode to the posterior edge of the fourth somite. The tissues were removed and placed in a 1.5 mL conical snap-cap plastic tube on ice and 1× enzyme digestion buffer containing 100 μL of *Bacillus licheniformis* stock (100 mg/mL, Sigma-Aldrich, P5380), 895 μL of DPBS, and 5 μL of 0.5 M EDTA. The tube was quickly shaken thoroughly to mix and resuspend, incubated in ice-water bath, and triturated using a cut-down 1 mL pipette tip for 15 seconds every 2 minutes. Cells were filtered through a 40 μm strainer, rinsed with 10 mL of ice-cold PBS/BSA, and weighed. Subsequently, 300 g of the cells were transferred to 15 mL conical tube, centrifuged at 300*g* for 5 minutes at 4°C, and the supernatant discarded. The pelleted cells were then resuspended in 100 μL of ice-cold DPBS and counted on a Countess II (Thermo Fisher Scientific). Cell count and viability were determined using trypan blue dye exclusion.

### QC.

QC was evaluated from the distribution of QC covariates in data set visualizations and clustering after multiple times of analyzing the data and comparison with bulk RNA-Seq data. The analysis showed that use of fraction of counts from mitochondrial genes per barcode was too stringent and resulted in significant loss of signal since cardiovascular progenitor cells and migrating NCCs had a high degree of mitochondrial content. A more permissive QC threshold based on only 2 QC covariates, count depth and the number of genes per barcode, was considered as more appropriate. Capture sites that were either empty or contained multiple cells were filtered. DoubletFinder (53) and high-count depth threshold were also used to exclude doublets. Normalization protocol included count depth scaling (AKA CPM normalization). Gene normalization, which constitutes scaling gene counts to have zero mean and unit variance (*z* scores), was applied. Normalized data were log-transformed for use with downstream analysis. Data correction for biological effects based on cell cycle signals was avoided because of the presence of a large number of proliferating cells.

### Single-cell sequencing pipeline.

Ten thousand cells per sample were used for the 10x Genomics Cell Ranger pipeline with a combined human genome and the ZsGreen1 gene to obtain count matrices for the WT and KO conditions. There were an average of 2742 and 3102 counts per cell or an average of 1294 and 1432 unique genes detected in WT and KO conditions, respectively. Only samples with RIN greater than 6.5 were used. Library preparations and sequencing were performed by the Yale Center for Genome Analysis. Briefly, gel beads in emulsion (GEMs) were created using Chromium Next GEM Single Cell 3′ Gel Beads v3.1 kit (10x Genomics) and barcoded. GEMs generated were used for cDNA synthesis and library preparation using the Chromium Single Cell 3′ Library Kit v3.1 (10x Genomics) and sequenced on NovaSeq 6000 system using HiSeq 100 base pair reads and dual indexing.

### ScRNA-Seq data alignment.

scRNA-Seq samples were demultiplexed using MULTI-Seq and barcoded and pooled on droplet microfluidic emulsion and sequenced with the 10x Genomics Chromium system. Cell Ranger software v1.3.1 was used for library demultiplexing, FASTQ file generation, read alignment, and unique molecular identifier (UMI) quantification. The resulting raw UMI counts in each cell were normalized to their library size. The normalized counts were square-root transformed. We then used a combination of 3 metrics to find differentially expressed genes across conditions: (a) the binary logarithm of fold change between mean counts; (b) the Wasserstein or earth mover’s distance (EMD); and (c) adjusted *P* value from a 2-sided Mann-Whitney *U* test with continuity and Benjamini-Hochberg correction, as described by Moon et al. (22). A *P* value less than or equal to 0.01 was used as the threshold for statistical significance. The top 30 differentially expressed genes (up- or downregulated, ranked by Wasserstein distance, were represented in heatmaps. To identify putative cellular functions changed across conditions, we performed PANTHER-GO gene set enrichment analysis for each cluster (54) using the default human PANTHER-GO reference list as a background.

### scRNA-Seq data analysis.

We used the standard scRNA-Seq analysis pipeline for clustering (55). We removed cells with less than that 200 and more than 6000 raw UMIs to account for doublets. Next, we removed genes that were expressed in fewer than 3 cells and removed cells that expressed fewer than 200 genes. We also removed UMIs pertaining to mitochondrial and ribosomal genes. The counts in each cell were normalized to their library size. Then, normalized counts were square-root transformed, similar to a log transform but not requiring addition of a pseudo count. Data preprocessing was performed in Python (v3.7.4) using Scanpy (v1.4.6) (56, 57).

We visually observed batch effects between conditions in 2D cellular embeddings. To remove these batch effects for downstream analysis, we used an approximate batch-balanced k nearest neighbor (kNN) graph for manifold learning (BB-kNN batch-effect correction) using Scanpy’s fast approximation implementation (56, 57). For each cell, the 3 nearest neighboring cells in each condition were identified by Euclidean distance in 100-dimensional principal component analysis (PCA) space. This kNN graph was used as the basis for downstream analysis.

To visualize the scRNA-Seq data, we implemented 2 nonlinear dimension reduction methods and used the BB-kNN batch-corrected connectivity matrix as input for uniform manifold approximation and projection (UMAP) (58) and PHATE (22). UMAPs were generated using a minimum distance of 0.5. PHATE projections were generated with a gamma parameter of 0.

For cell clustering, we used the Louvain community detection method (59) with the BB-kNN graph. We used high-resolution community detection and merged clusters based on expression of hand-picked markers for the cell types (Supplemental Figure 13). In order to minimize the effect of dropout for clustering, we imputed counts using Markov affinity-based graph imputation of cells (MAGIC) (60).

To find the differentially expressed genes between the WT and KO conditions in each cell type, we used 3 metrics: the 1-dimensional Wasserstein or EMD, an adjusted *P* value from a 2-sided Mann–Whitney *U* test with continuity and Benjamini-Hochberg correction, and the binary logarithm of fold-change between mean counts. Significance was set to an adjusted *P* value of 0.01. The EMD can be defined as the minimal cost to transform distribution to another and has been previously used to perform differential gene expression analysis (61–63).

### O9-1 cell culture and differentiation and target gene KO in O9-1 cells.

O9-1 cells were obtained from MilliporeSigma. Low-passage O9-1 cells (SCC049) were maintained on Matrigel-coated dishes in complete embryonic stem cell medium with 15% fetal bovine serum and leukemia inhibitory factor (ES-101-B) supplemented with bFGF.

The O9-Cas9 cell line was generated by transducing the O9-1 cells with lentivirus, which packaged an EFS-Cas9-T2A-blasticidin expression cassette; polybrene was used for improving virus infection efficiency. One day after lentiviral transduction, 10 μg/mL blasticidin was used for selection for 5 days to get a pure O9-Cas9 cell line. SgRNAs were designed (AACGGGGAGTGCCCCATGCA) and cloned into a backbone plasmid, U6-sgRNA-EFS-puromycin, and then packaged into lentivirus. The puromycin selection (10 μg/mL) was performed after lentiviral transduction for 3 days. The T7E1 assay was performed at day 7 after sgRNA lentiviral transduction to evaluate target gene KO efficiency.

### Cell cycle distribution (Hoechst/pyronin Y).

Single-cell suspensions were prepared and adjusted to contain 2 × 10^6^ cells/mL. Single-cell suspensions were stained with Hoechst (10 μg/mL) and pyronin Y for 15 minutes at 37°C.

### Statistics.

In vivo studies included a minimum of 3 mice in each group. In vitro studies were carried out in more than 3 independent experiments. The comparison between different groups was done by a 2-tailed unpaired *t* test. The normality was tested by Kolmogorov-Smirnov test. An *F* statistic was calculated to determine whether variances were different between samples, and the *P* values were then corrected with Welch’s 2-tailed *t* test. The comparisons between multiple groups were done by 1-way ANOVA. A Mann-Whitney test was conducted for non-normally distributed data. Fisher’s exact test was carried out for the continuous variables. The preparation of graphs and all statistical analyses, including 2-tailed Student’s *t* tests, 2-way ANOVA (SigmaPlot), and testing for equal variance, were carried out using GraphPad Prism 8.1 Project software. *P* values less than 0.05 were considered significant. Data are presented as mean ± SEM. Fluorescence images were evaluated using ImageJ (NIH).

### Code and data availability.

All differential gene expression analyses from bulk and scRNA-Seq and their associated metrics have been deposited in NCBI’s Gene Expression Omnibus (GSE195590).

### Study approval.

Animal procedures were conducted according to an approved protocol by the Yale University IACUC.

## Author contributions

LH designed and conducted experiments, acquired data, analyzed data, and helped with preparing the manuscript. NL generated floxed mice. VG analyzed scRNA-Seq data. SM analyzed bulk RNA-Seq data. LY, YW, and JL analyzed data. AG and JR provided the mice. KKH and AE supervised the data analysis. CH helped with the writing of the manuscript and provided instruction on the preparation of the data. DVD supervised the analysis of scRNA-Seq data. AM designed and supervised the research studies and wrote the manuscript.

## Supplementary Material

Supplemental data

## Figures and Tables

**Table 2:**
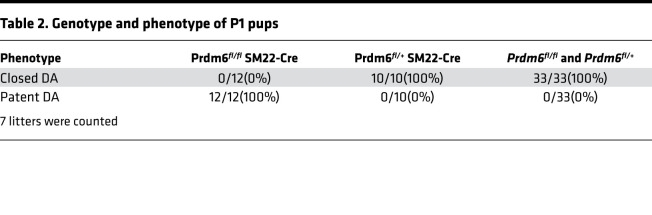
Genotype and phenotype of P1 pups

**Table 1:**
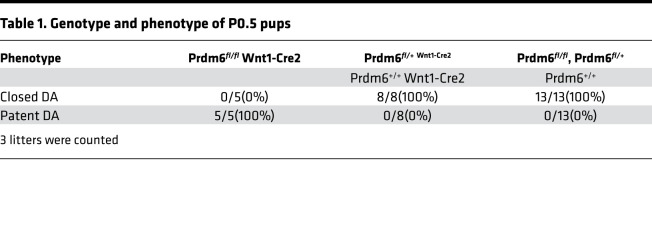
Genotype and phenotype of P0.5 pups

## Version 1 02/02/2022

In-Press Preview

## Version 2 02/22/2022

Electronic publication

## Version 3 03/11/2022

Updated supplemental data
