# Supplementary material for: Prdm6 controls heart development by regulating neural crest cell differentiation and migration
Source: JCI Insight. 2022 Feb 22;7(4):e156046. doi: 10.1172/jci.insight.156046 (PMC8876496; doi:10.1172/jci.insight.156046)
Supplement: Supplemental data [file jciinsight-7-156046-s013.pdf]

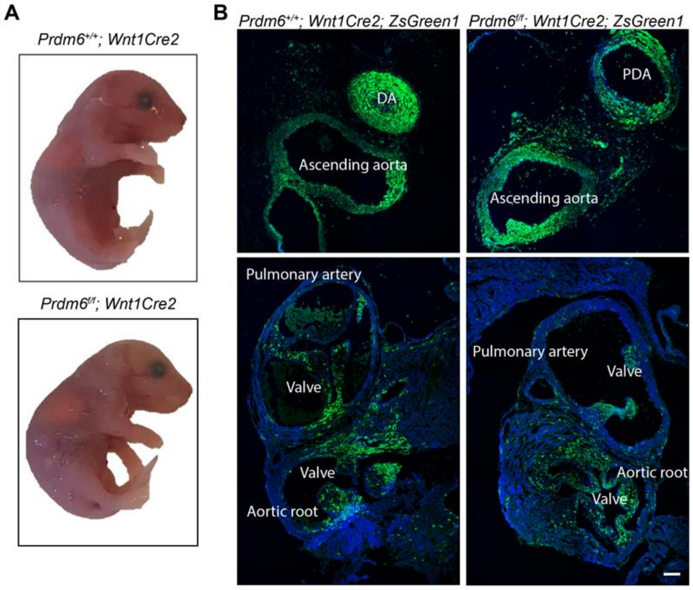

Supplemental Figure 1

(**A**) The gross appearance of control (top) and *Prdm6$^{f/f}$; Wnt1-Cre2* (bottom) pups at P0.5. (**B**) The confocal images of representative cross sections of the ascending aorta, DA (top) and semilunar valves (bottom) in *Prdm6$^{f/f}$; Wnt1-Cre2; ZsGreen1* (right) and corresponding littermates (left) at P0.5 demonstrating ZsGreen1 positive cells(green). DAPI is shown in blue. Scale bar, 50 µm.

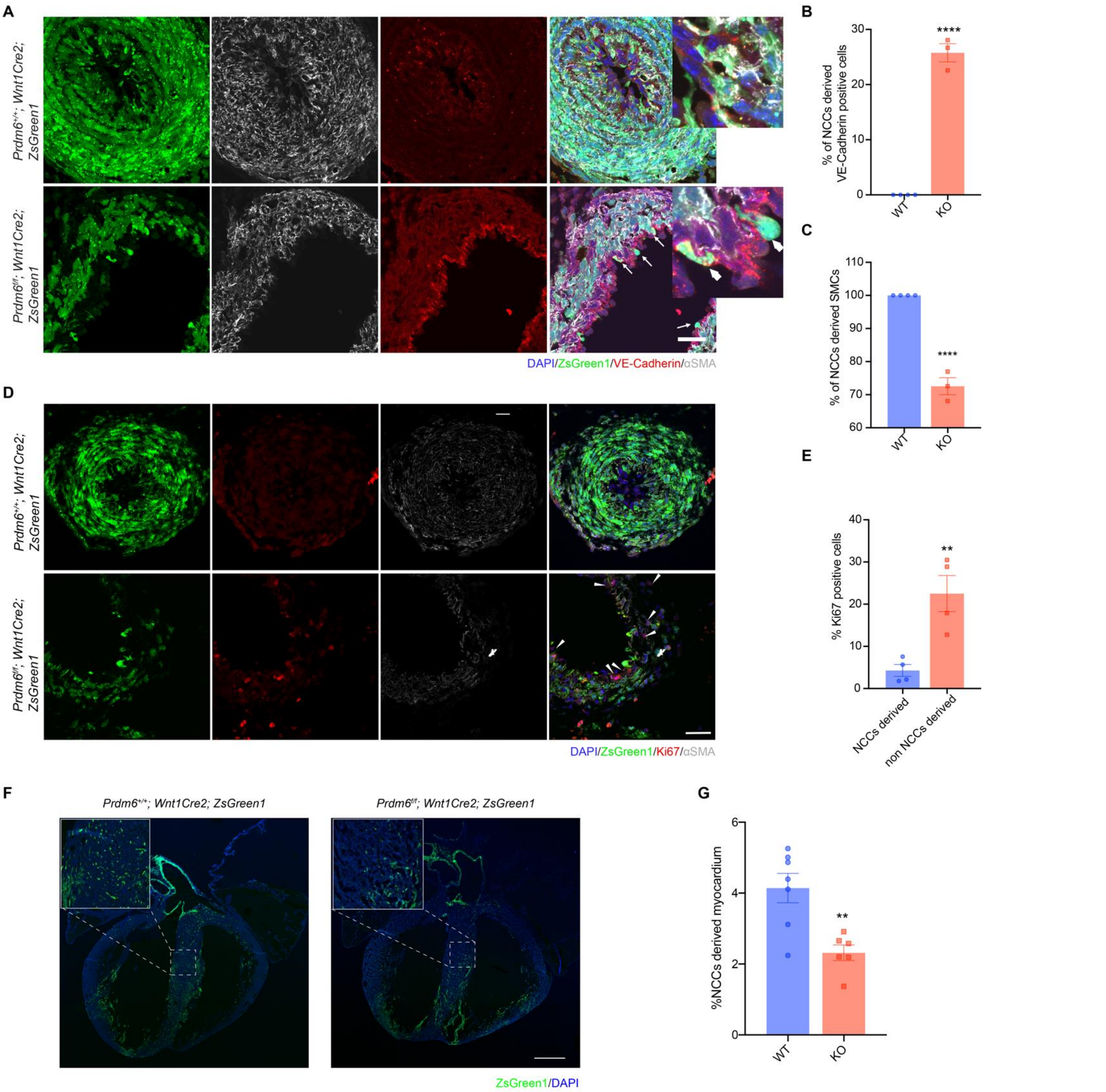

Supplemental Figure 2

(**A**) Representative cross sections of DA of wildtype (top) and *Prdm6f/f; Wnt1-Cre2; ZsGreen1*(bottom) DA at P 0.5 by confocal imaging, demonstrating the colocalization of αSMA (grey) and ZsGreen1(green). In contrast to controls only a fraction of SMCs of *Prdm6f/f; Wnt1-Cre2; ZsGreen1* are NCC derived. In addition, some NCC-derived cells stained positively for VE-Cadherin (red, white arrows), suggesting altered fate CNCCs. Scale bar, 20 µm. (**B** and **C**) Percent neural crest cell derived VE Cadherin positive cells and SMCs. (**D**) The confocal images of representative cross sections of DA of control (top) and *Prdm6f/f; Wnt1-Cre2; ZsGreen1*(bottom) at P0.5 stained for Ki67(red) and

αSMA (grey) show the localization of Ki67 and αSMA in ZsGreen1 (green) negative cells, indicated by white arrowhead. (**E**) Quantification of Ki67 positive cells. (**F**) Frontal section through the heart at P0.5 demonstrating the presence of ZsGreen1 positive cells(green) in the ventricular septum and to a lesser degree in the innermost layer of the left and right ventricles in wildtype control mice (left); the insets show the magnified regions of the intraventricular septum. Scale bar, 200 µm. (**G**) Quantification of NCC derived myocardium. There were significantly fewer ZsGreen1 positive cells in the heart of *Prdm6f/f; Wnt1-Cre2; ZsGreen1* mice (right). The image intensities were quantified by ImageJ, the thresholds for positive color detection were kept constant between different images. Each dot represents a biological replicate. The comparison between different groups was done by a 2-tailed unpaired t-test and data is shown as mean ± s.e.m. *** P<0.01, **** P<0.0001.* All controls were the corresponding littermates (n=4-7 per group).

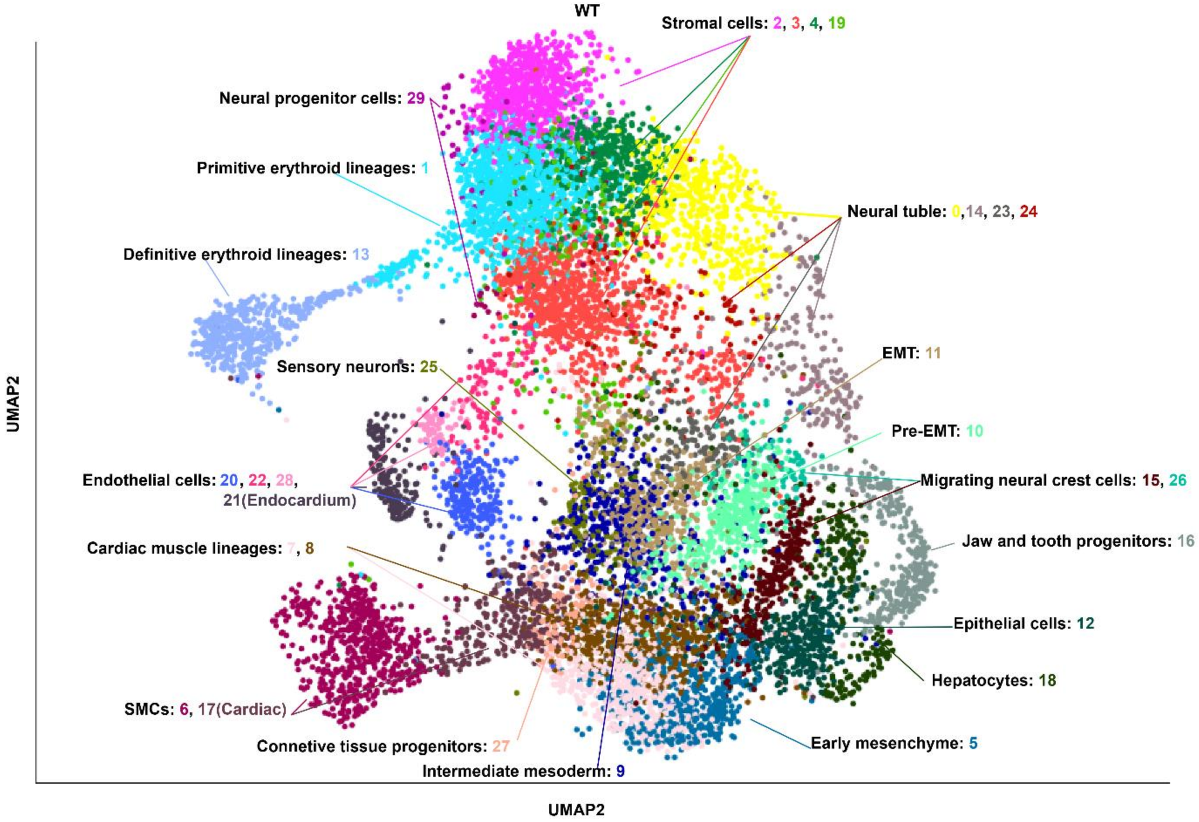

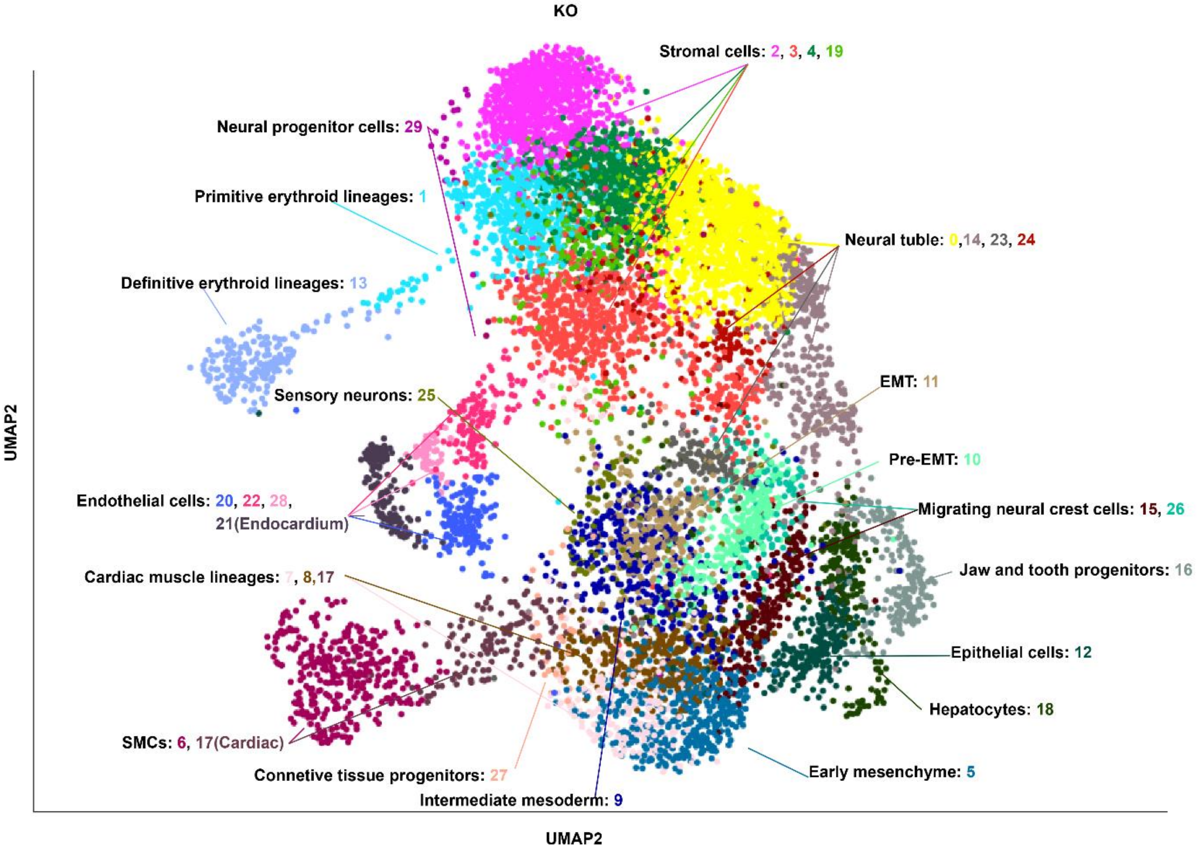

Supplemental Figure 3

UMAP figures of scRNA-seq data of cardiac neural crest region of *Prdm6$^{f/f}$; Wnt1-Cre2; ZsGreen1* mice and corresponding littermates demonstrating different clusters and corresponding cell counts.

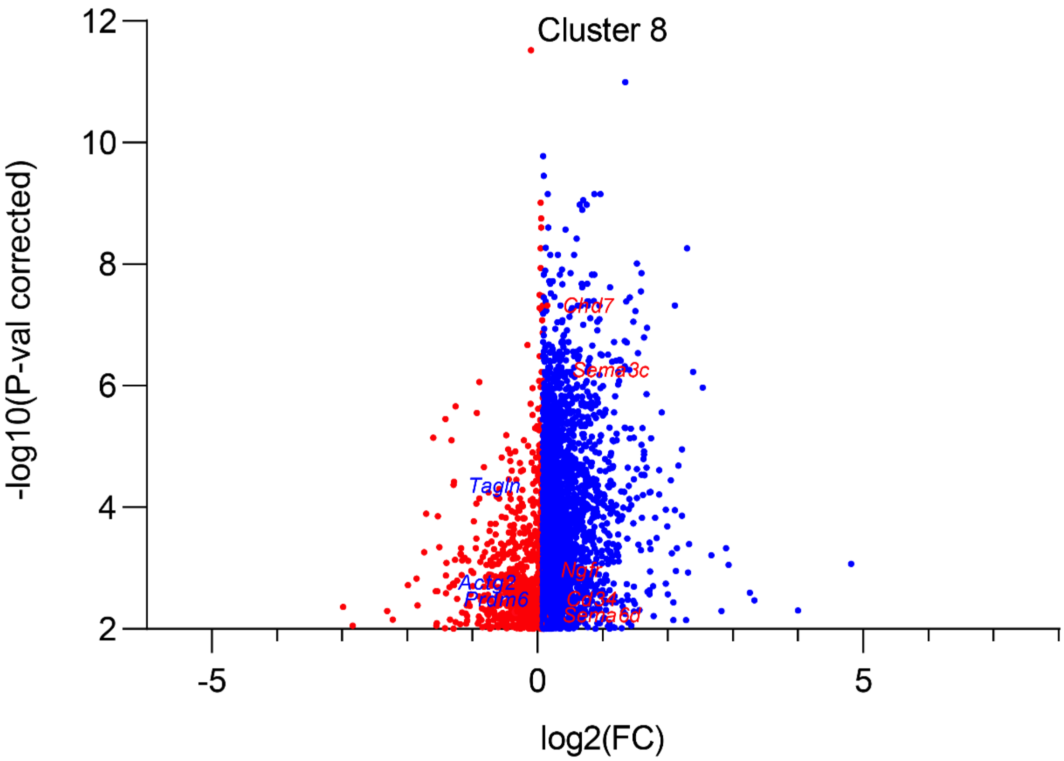

Supplemental Figure 4

The volcano plot of cluster 8 from scRNA-seq data of *Prdm6$^{f/f}$; Wnt1-Cre2; ZsGreen1* mice vs. corresponding littermates at E9.5. The blue dots indicates genes that are upregulated and red genes that are downregulated in *Prdm6$^{f/f}$; Wnt1-Cre2; ZsGreen1* vs. wildtype littermates.

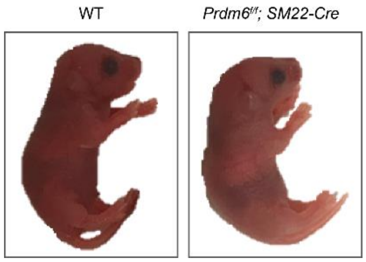

Supplemental Figure 5

The gross appearance of control (left) and *Prdm6$^{f/f}$; SM22-Cre* mice (right) pups at P0.5.

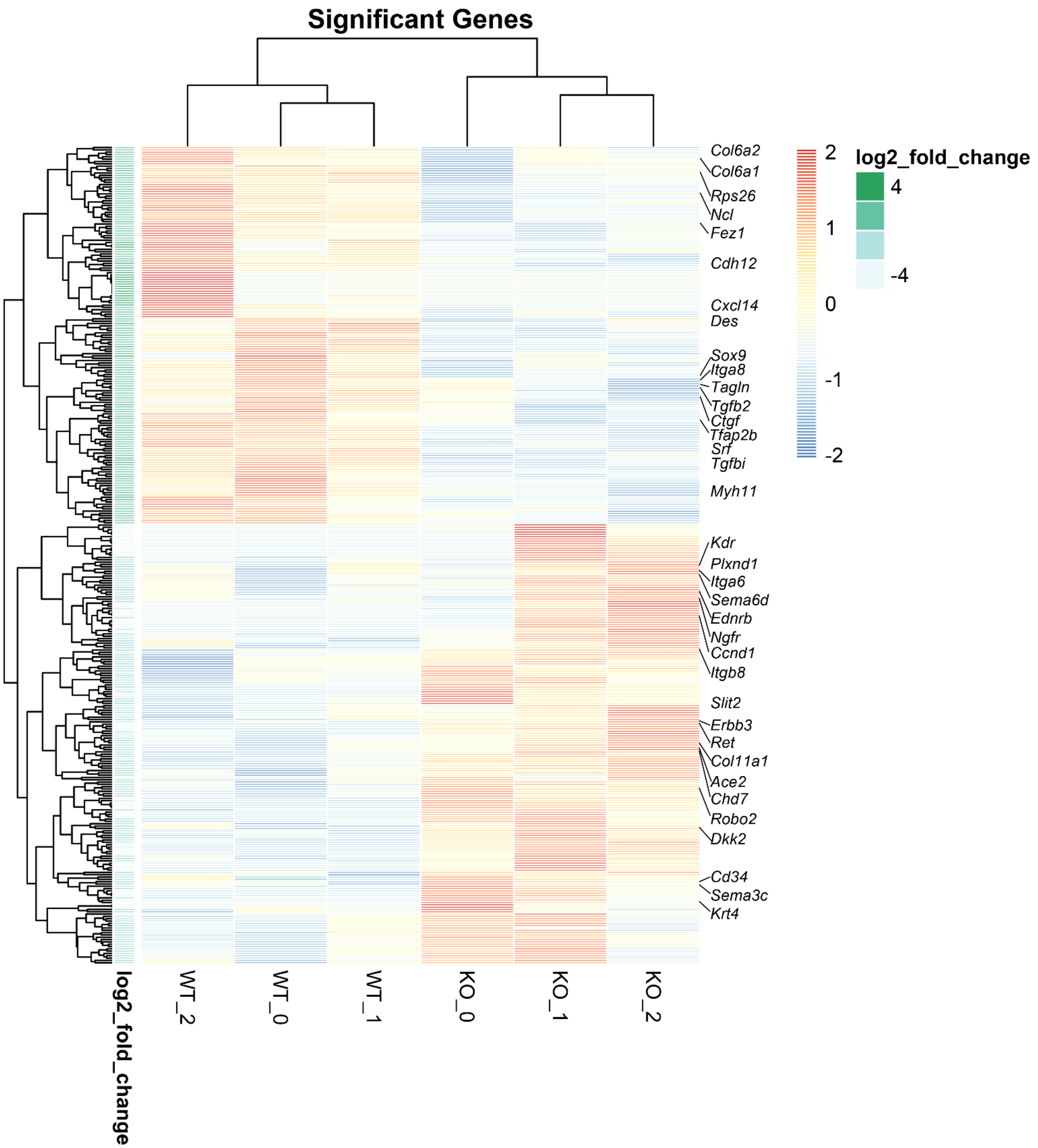

Supplemental Figure 6

The heatmap of upregulated (bottom half) and downregulated (top half) genes from bulk RNA-seq data of *Prdm6$^{f/f}$; SM22-Cre* mice DA compared to corresponding littermate controls at E17.5.

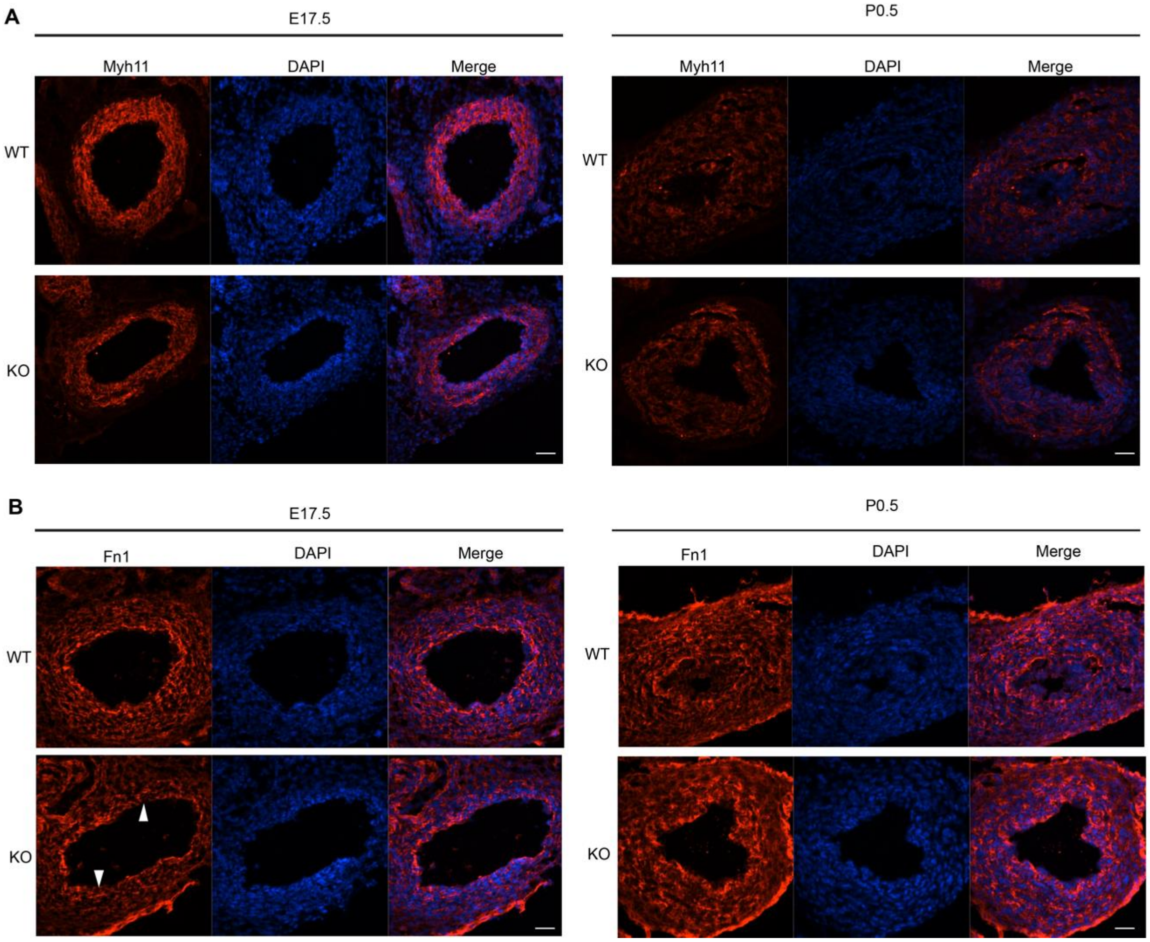

Supplemental Figure 7

(**A**) The confocal images of representative cross sections of DA of wildtype littermates (top) and *Prdm6$^{f/f}$; SM22-Cre* embryos (bottom) at E17.5 and P0.5. Immunostained for Myh11(red) and co-stained with DAPI (blue). Scale bar, 50 µm. (**B**) The confocal images of representative cross sections of DA of *Prdm6$^{f/f}$; SM22-Cre* embryos (bottom) and corresponding littermates (top) and at E17.5 and P0.5. Immunostained for Fn1(red) and costained with DAPI (blue). Scale bar, 50 µm.

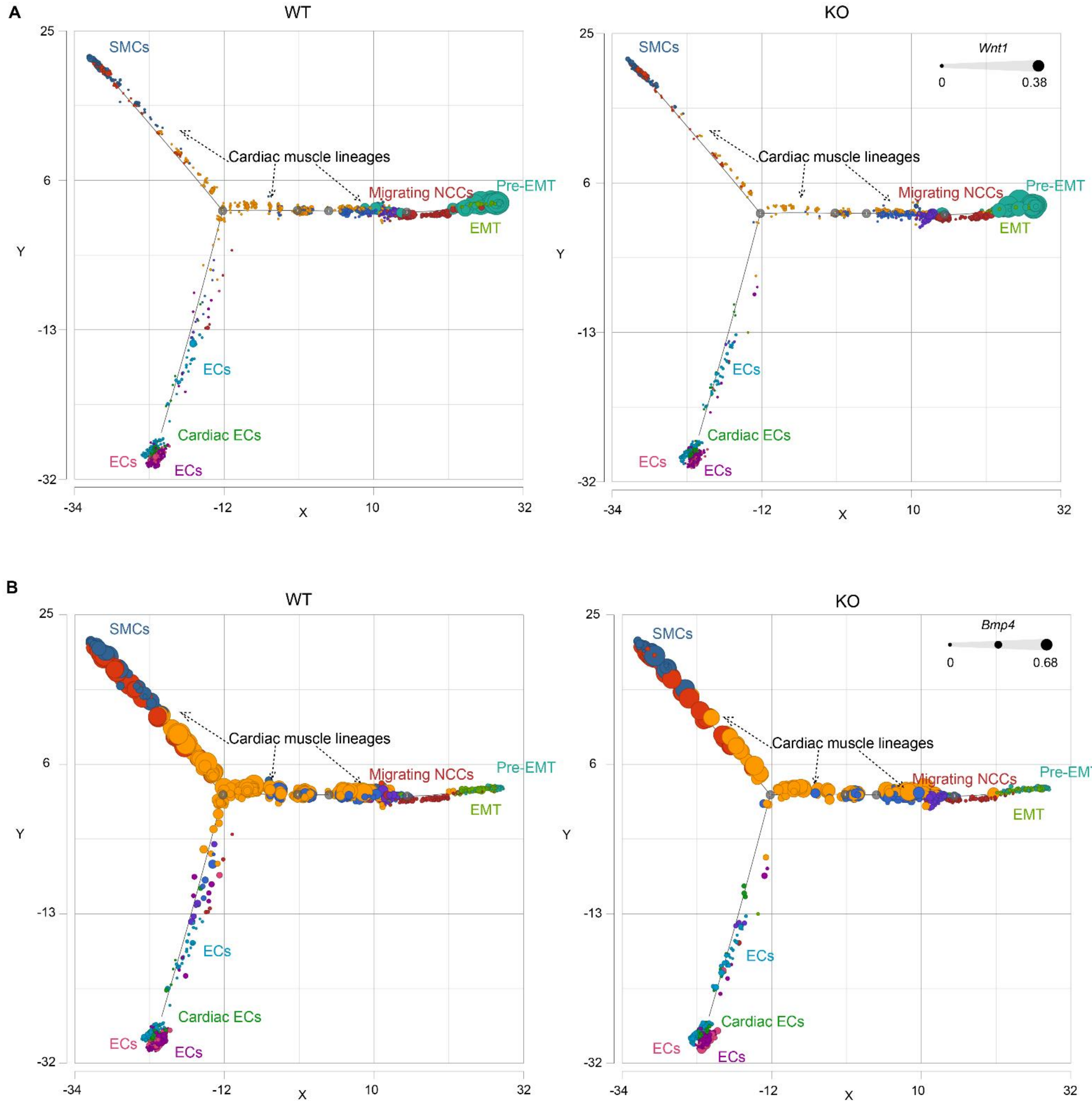

Supplemental Figure 8

(**A** and **B**) The Trajectory inference of *Wnt1* (**A**) and *Bmp4*(**B**) transcripts in different cell clusters. pre-EMT, EMT, migrating neural crest cells and cardiac muscle lineages are highlighted in the trajectory. Also, Labeled are smooth muscle and endothelial cells. The expression levels of *Wnt1*(**A**) and *Bmp4*(**B**) are represented by the sizes of the circles.

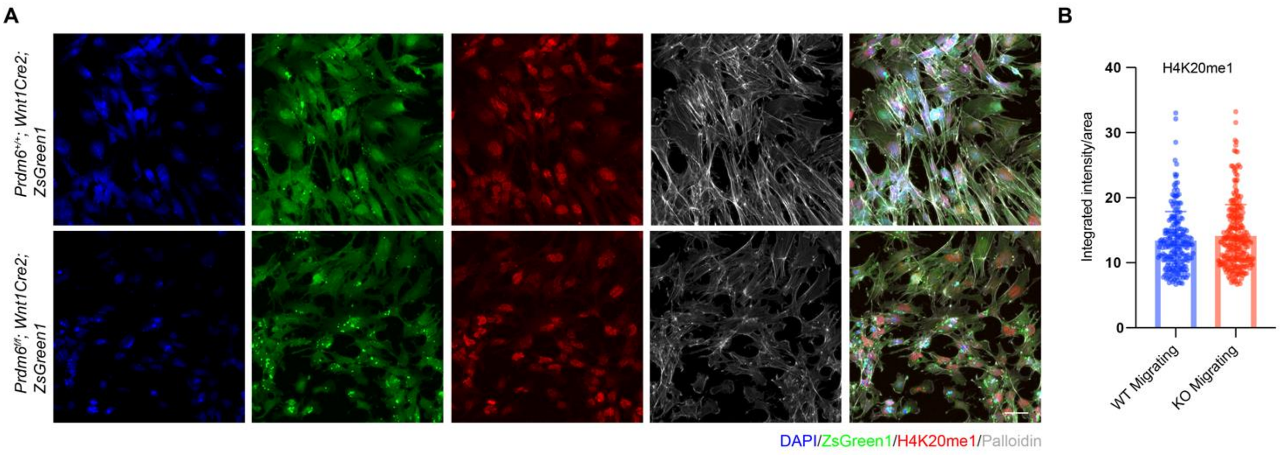

Supplemental Figure 9

(**A**) H4K20 mono-methylation (red) of NCCs in the migrating cells of *ex-vivo* cultured neural tubes of wildtype (*Prdm6*$^{+/+}$*; Wnt1-Cre2; ZsGreen1*) and knockout (*Prdm6*$^{f/f}$*; Wnt1-Cre2; ZsGreen1*) mice at E9.5 stained with DAPI (blue), and phalloidin (grey). Scale bar, 50 µm. (**B**) Quantification of H4K20me1. Data are mean ± s.e.m. For statistical analysis, groups were compared using unpaired *t*-test. Each dot represents a cell. The image intensities were quantified by ImageJ, the thresholds for positive color detection were kept constant between different images.

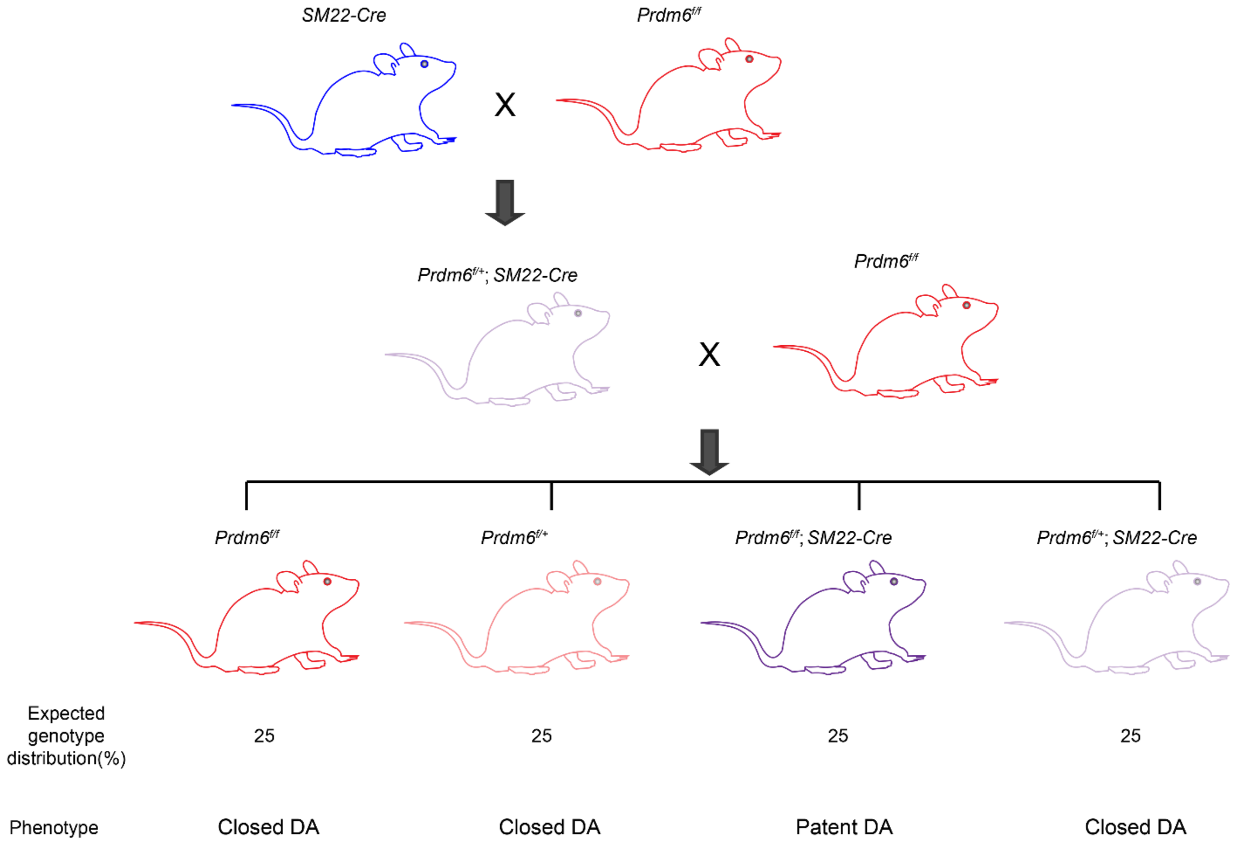

Supplemental Figure 10

The schematic of crossbreeding of *Prdm6*$^{f/f}$ with *SM22-Cre* mice.

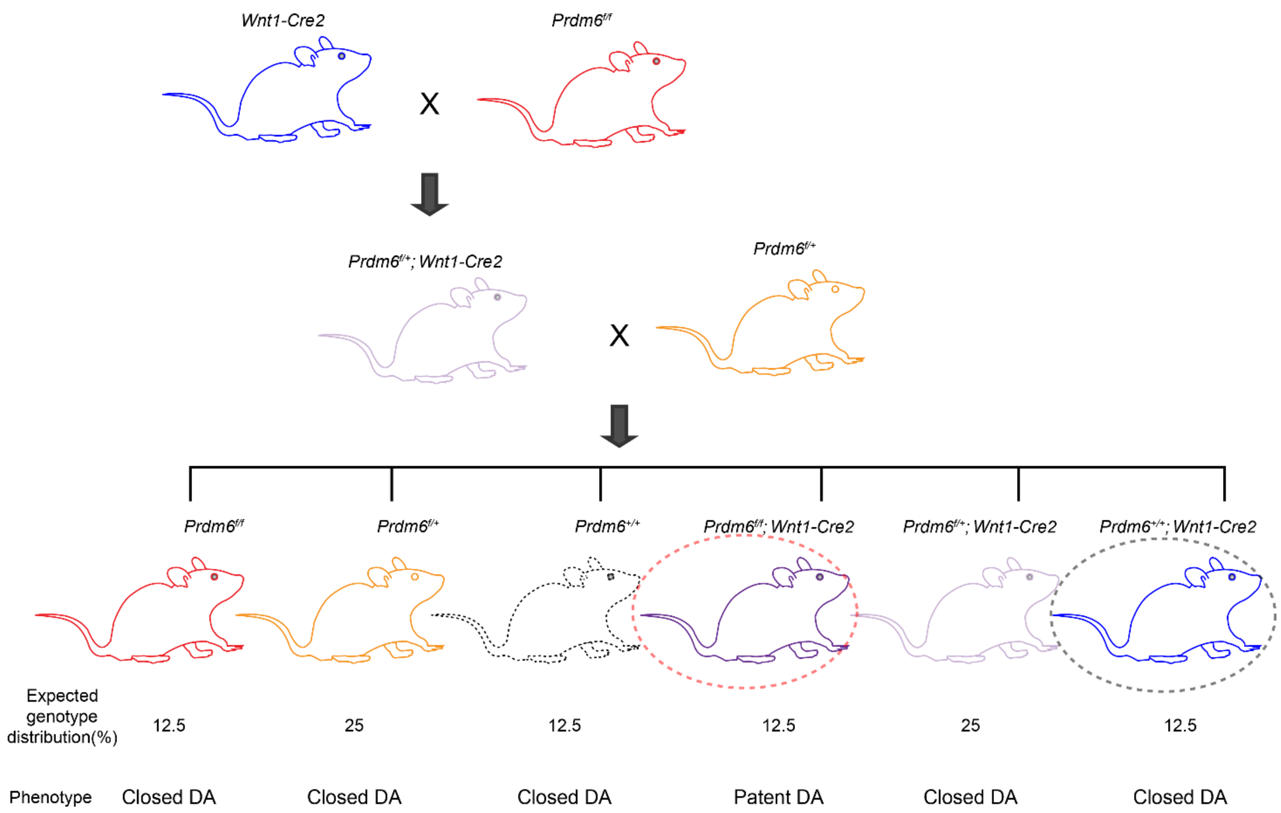

Supplemental Figure 11

The schematic of crossbreeding of *Prdm6*$^{f/f}$ with *Wnt1-Cre2* mice.

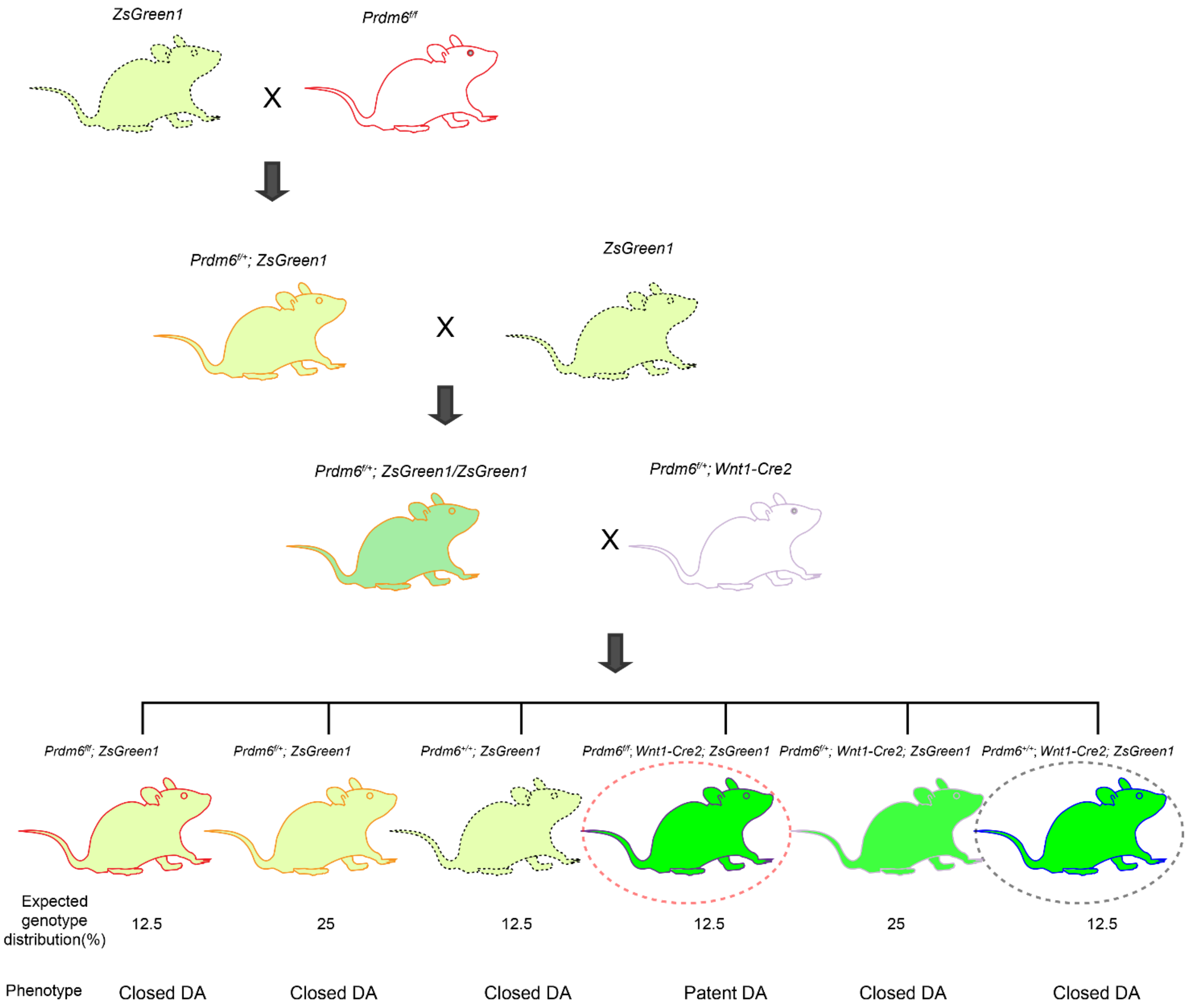

Supplemental Figure 12

The schematic of crossbreeding of *Prdm6$^{f/f}$* with *ZsGreen1* mice and *Wnt1-Cre2* mice.

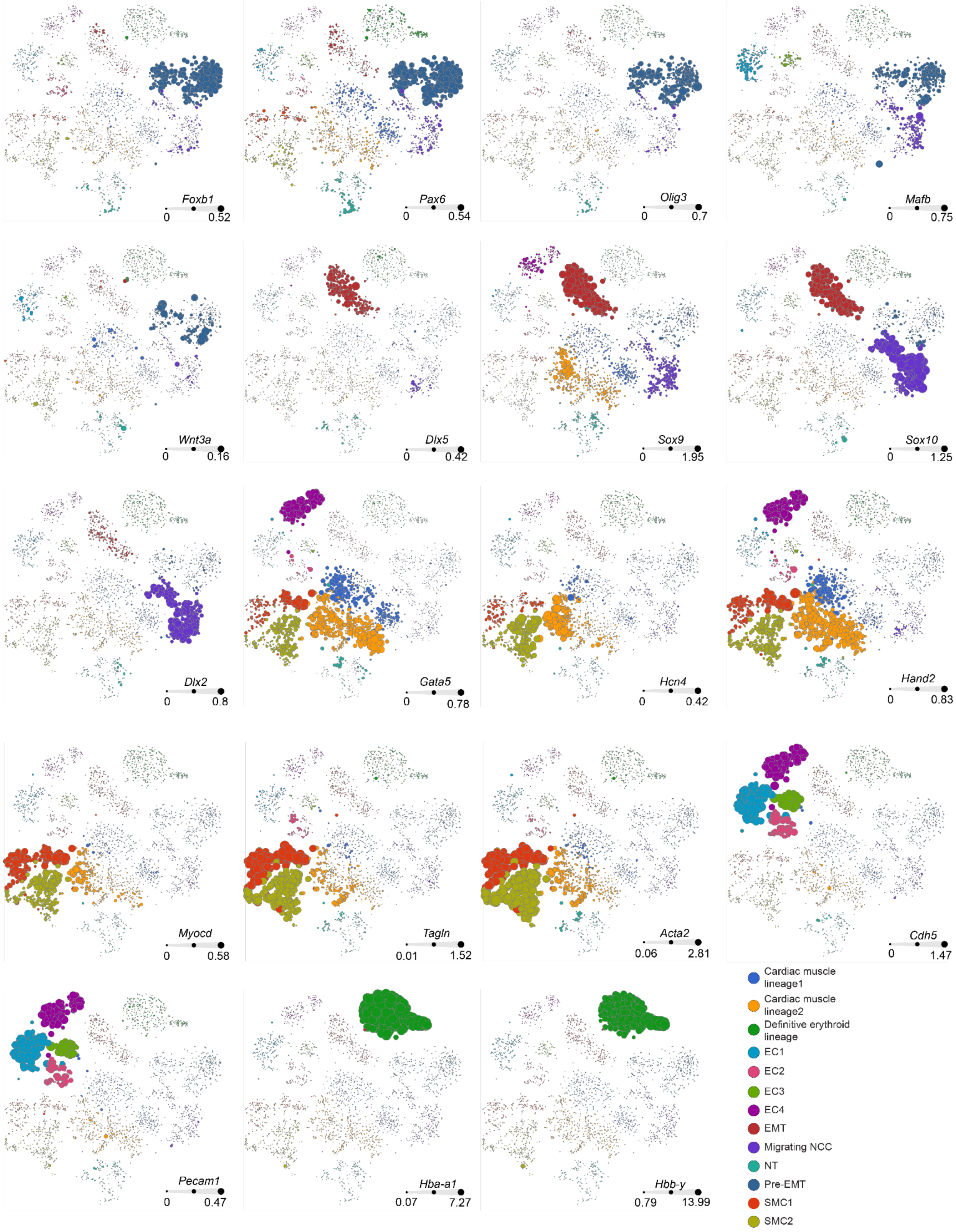

Supplemental Figure 13

The marker genes used for the annotation of cell types in scRNA-seq clusters of wildtype mice.

Table S1 Markers used for cell type identification

Cell type	Markers used for cell type identification
Neural tube	Foxb1, Scube2, Slc2a1, Pax6, Itgb8, Olig3
Pre-EMT	Mafb, Wnt3a, Msx1
EMT	Sox9, Dlx5, Pdgfra, Pak3
Migrating Neural crest	Sox10, Ret, Dlx2
Endothelial cells	Pecam1, Cdh5
Endocardium	Pecam1, Cdh5, Tbx20
Smooth muscle cells	Acta2, Tagln
Cardiac smooth muscle cells	Acta2, Tagln, Hand2
Definitive erythroid lineage	Snca, Hba-a1, Hbb-y
Primitive erythroid lineage	Hba-a1, Hbb-y
Cardiac muscle lineage	Hcn4, Gata5, Hand2, Myocd
Myocytes	Neb, Myh3
Epithelial cells	Epcam, Trp63, Grhl2
Jaw and tooth progenitor	Pax9, Col2a1, Foxp2
Hepatocytes	Afp, Alb
Sensory Neurons	Neurod1, Neurod4, Dll1
Early Mesenchyme	Twist1, Prrx1, Prrx2

Table S2
List of genes with altered expression in RNA-seq of *Prdm6* $^{f/f;}$ *SM22-Cre* mice DA compared to wildtype littermates

Downregulated genes				Upregulated genes			
gene_id	KO	WT	KO/WT	gene_id	KO	WT	KO/WT
Gna14	1.11141	17.6556	0.0629	Mycbp2	20.4282	12.4219	1.6445
Clca2	20.2606	204.215	0.0992	Tns3	45.9269	27.3543	1.6790
Cdh12	0.67666	4.80726	0.1408	Akap6	18.5028	10.7544	1.7205
F3	1.91059	13.2167	0.1446	Cd93	61.5216	35.5536	1.7304
Fez1	4.90425	29.7111	0.1651	Lhfpl2	46.4785	26.7589	1.7369
Nell1	3.63063	19.5059	0.1861	Trib2	36.1118	20.7205	1.7428
Akap5	3.22744	16.4713	0.1959	Lama4	86.3094	49.2869	1.7512
Hspb6	5.65519	27.1475	0.2083	Plxnd1	33.0344	18.8395	1.7535
Igfbp2	12.3257	59.0911	0.2086	Bnip3l	115.563	65.6096	1.7614
Tgfbi	13.354	63.683	0.2097	Ube2l6	125.813	71.1019	1.7695
Edf1	11.487	54.4791	0.2109	Abca1	20.12	11.3548	1.7719
1700021F(	7.73667	36.104	0.2143	Stard13	19.092	10.6582	1.7913
Stac	2.76153	12.2999	0.2245	Sema3c	114.86	63.9901	1.7950
Tpm2	55.8774	248.521	0.2248	Pcdh9	22.5985	12.5212	1.8048
Ptger4	6.07377	25.6448	0.2368	Gpr125	34.4939	19.0567	1.8101
Slc24a4	6.54277	27.4936	0.2380	Psme3	42.2755	23.3397	1.8113
Pi16	8.48832	35.5035	0.2391	Flrt2	42.3776	23.33	1.8164
Lars2	33.9031	136.745	0.2479	Bach1	18.1204	9.96908	1.8177
Tnmd	4.82798	19.0162	0.2539	Slit2	38.4655	20.8264	1.8470
Tmsb10	51.5224	200.155	0.2574	Thrb	20.6604	11.1224	1.8575
Lmod1	16.0025	61.4054	0.2606	Abca8a	27.7216	14.9215	1.8578
Sh3bgr	10.525	40.3014	0.2612	Dkk2	41.7847	22.3739	1.8676
Tfap2b	1.79974	6.72904	0.2675	Gdf10	51.3826	27.4685	1.8706
Myh11	123.264	446.745	0.2759	Capn6	77.6762	41.4996	1.8717
Dpt	40.6297	146.197	0.2779	Cacna2d1	15.2021	8.10705	1.8752
Sox9	2.00719	7.19526	0.2790	Tmod2	13.5389	7.06568	1.9161
Apcdd1	5.83425	20.8352	0.2800	Unc13b	14.9149	7.69526	1.9382
Kcne4	7.58147	26.9431	0.2814	Sema6d	20.7419	10.6581	1.9461
Leo1	5.44866	19.1296	0.2848	Kdm7a	12.7107	6.51811	1.9501
Upf3a	6.39863	22.325	0.2866	Dcaf12	28.3763	14.3939	1.9714
Nov	30.5554	100.711	0.3034	Cd34	303.162	150.032	2.0206
Shfm1	146.869	482.257	0.3045	Lama3	7.94038	3.90386	2.0340
Ppp1r14a	34.4151	107.359	0.3206	Nrp2	29.0161	14.2119	2.0417
Actg2	143.548	445.126	0.3225	Palmd	36.4085	17.8269	2.0423
Hspa2	5.34857	16.5301	0.3236	Itga6	28.5004	13.894	2.0513
Dctn6	15.6237	48.1418	0.3245	Thbd	53.3246	25.9853	2.0521
Rpl37	114.524	348.507	0.3286	F11r	24.4916	11.8352	2.0694
Itga8	28.6671	86.2129	0.3325	Cd24a	138.942	66.2627	2.0968
Synpo2	20.892	62.5879	0.3338	Hmgcs1	29.081	13.8496	2.0998

Nt5e	3.09203	9.25377	0.3341	Nbea	37.7549	17.9454	2.1039
Shroom3	4.67613	13.8604	0.3374	Zfp329	7.74488	3.66472	2.1134
Zcrb1	15.057	42.5902	0.3535	Lum	154.032	72.8065	2.1156
Egln3	10.214	28.6217	0.3569	Fam101b	17.3789	8.08894	2.1485
Osr1	19.3164	53.52	0.3609	Unc5c	13.5224	6.20242	2.1802
Ndufa6	41.064	112.378	0.3654	Col12a1	197.222	90.1754	2.1871
Ctgf	96.1058	260.585	0.3688	Snca	158.506	71.7893	2.2079
Mrpl42	31.4593	84.7988	0.3710	Kif1a	18.0514	8.16546	2.2107
AI414108	5.19794	14.0084	0.3711	Bpgm	117.502	52.6147	2.2333
Khdrbs3	10.3375	27.6951	0.3733	Fam220a	44.0955	19.5579	2.2546
Alyref	28.7233	75.6023	0.3799	Ndrg1	36.9189	16.3574	2.2570
Clec3b	24.1913	63.3336	0.3820	Fech	94.9287	41.9225	2.2644
Lgr6	12.416	32.5015	0.3820	Slc26a7	31.137	13.7238	2.2688
Ccdc55	6.83497	17.8414	0.3831	Mkrn1	56.9541	24.8432	2.2925
Galnt16	18.8034	48.0167	0.3916	Ube2o	58.3046	25.2284	2.3111
Lrrc75b	6.18042	15.5627	0.3971	Chd7	5.56373	2.40548	2.3129
Vav3	4.5086	11.3097	0.3986	Cttnbp2	8.46281	3.647	2.3205
Chmp5	17.6036	43.922	0.4008	Lyz2	105.343	44.9659	2.3427
Des	20.2523	50.3634	0.4021	Ngfr	18.8331	8.02666	2.3463
Nexn	22.8898	56.6897	0.4038	Epb4.1	185.638	78.7342	2.3578
D8Ertd738	30.5977	75.4975	0.4053	Kdr	27.5996	11.6188	2.3754
Sumo1	31.6556	78.0988	0.4053	Ccp110	13.9105	5.74433	2.4216
Clca1	11.4423	28.1994	0.4058	Gucy1a2	4.77864	1.9684	2.4277
Chmp2b	28.5029	69.1688	0.4121	Gja4	49.0436	20.1911	2.4290
Col24a1	15.6664	37.7809	0.4147	Peli2	10.233	4.19588	2.4388
Rpl23a	326.473	782.221	0.4174	Piezo2	27.398	11.2058	2.4450
Utp3	16.8773	39.9905	0.4220	Col11a1	21.0153	8.5288	2.4640
Rpl21	108.123	253.916	0.4258	Ets1	26.711	10.7874	2.4761
Palm2	7.1344	16.7333	0.4264	Arhgap26	6.28899	2.52945	2.4863
Igsf9b	6.31517	14.7966	0.4268	Ccnd1	20.7864	8.30356	2.5033
Pdap1	14.4758	33.5203	0.4319	Fgf10	18.9307	7.52114	2.5170
Hist1h4d	204.326	469.741	0.4350	Atp2a3	11.5543	4.58086	2.5223
2810004N	19.8425	45.317	0.4379	Mmp15	11.3555	4.47878	2.5354
Chmp2a	27.7126	62.5001	0.4434	Hbb-bt	2324.8	909.562	2.5560
Itga9	28.5133	64.2544	0.4438	S1pr3	17.4535	6.82459	2.5574
Cthrc1	51.2625	114.899	0.4462	Stmn2	29.9605	11.6832	2.5644
Sulf2	51.6336	113.95	0.4531	Cd82	51.3744	20.0309	2.5648
Adamts2	35.3523	77.5955	0.4556	E2f2	10.9608	4.19476	2.6130
Fgfr2	13.8902	30.4773	0.4558	Dock10	17.3528	6.63811	2.6141
Ace	41.5671	89.7588	0.4631	Wipf2	8.48367	3.2211	2.6338
Sfrp2	396.969	854.257	0.4647	Kif21a	7.27371	2.7615	2.6340
Kcnj8	14.6741	31.207	0.4702	Hmgcr	24.9711	9.29311	2.6871

Lrrfip1	38.1289	81.0401	0.4705	Arhgef15	10.8453	3.87497	2.7988
Pclo	2.0559	4.35909	0.4716	Itgb8	17.4381	6.18292	2.8204
Rpl31	156.727	331.381	0.4730	Tbx2	12.2975	4.35886	2.8213
Ncl	41.2068	87.0477	0.4734	Alas2	585.881	205.05	2.8573
Myl12a	67.7796	141.828	0.4779	Flt4	11.5414	3.99286	2.8905
Mfap2	51.8995	108.468	0.4785	Cxcl13	88.0447	30.2288	2.9126
Hspb1	42.6243	88.9744	0.4791	Spta1	11.165	3.83194	2.9137
Rpl36a	210.539	439.017	0.4796	Itga2b	11.4442	3.8659	2.9603
Dmpk	22.7569	47.3354	0.4808	Fgl2	23.0803	7.72749	2.9868
Sdc4	20.2466	41.9695	0.4824	Lphn3	23.5686	7.83186	3.0093
Tagln	450.781	931.865	0.4837	Kcna6	9.50864	3.12561	3.0422
Qsox1	15.3056	31.4789	0.4862	Pnpo	34.4236	11.2967	3.0472
Abi3bp	24.8899	50.9892	0.4881	Mmrn1	45.861	14.9945	3.0585
Tcf7l1	16.3712	33.5277	0.4883	Slc25a37	131.91	43.0724	3.0625
Ybx1	150.527	301.885	0.4986	Asxl3	4.54493	1.4481	3.1385
Ltbp2	27.3218	54.5504	0.5009	Cldn5	38.3453	12.054	3.1811
Ngfrap1	79.6717	158.549	0.5025	Robo2	5.05965	1.58209	3.1981
Nkd1	9.361	18.5702	0.5041	Zfhx4	6.78811	2.12172	3.1993
Fndc1	34.9617	68.7135	0.5088	Hbb-b1	2230.38	686.05	3.2510
Csrp2	236.501	464.384	0.5093	Gyg	34.2801	10.4803	3.2709
Loxl1	47.543	92.8345	0.5121	Gpr126	10.5973	3.20896	3.3024
Cd248	66.009	128.087	0.5153	Ednrb	52.8307	15.5718	3.3927
Thumpd1	16.2225	31.4034	0.5166	Abca8b	8.63453	2.53406	3.4074
Pnn	19.5349	37.8155	0.5166	Cytl1	47.035	13.7562	3.4192
Tsnax	21.55	41.7161	0.5166	Fam46c	107.235	31.0726	3.4511
Srf	11.1145	21.47	0.5177	Scn7a	8.60211	2.43876	3.5272
Snrnp70	40.7273	78.3384	0.5199	Cadm1	13.0938	3.69563	3.5430
Pacsin2	21.7486	41.8254	0.5200	Tubb1	15.5809	4.32667	3.6011
Col6a2	106.183	202.938	0.5232	Gypa	36.236	10.0345	3.6111
Atp5f1	60.3675	114.817	0.5258	Atp8a1	5.74402	1.56292	3.6752
Serpinb6b	35.069	66.5442	0.5270	Ccdc3	25.8442	6.99831	3.6929
Tnxb	17.5465	33.2716	0.5274	Susd5	22.6979	6.12889	3.7034
Mfap4	153.781	290.404	0.5295	Slc4a1	62.0259	16.6608	3.7229
Rpl36	211.342	397.746	0.5313	Deptor	3.58506	0.91626	3.9127
Thbs1	72.4987	135.368	0.5356	Chl1	5.01961	1.27577	3.9346
Uqcrb	157.357	292.42	0.5381	Ace2	6.77037	1.70811	3.9637
Rps26	209.604	387.976	0.5402	Col8a1	9.70993	2.43676	3.9848
Mrfap1	88.4779	163.739	0.5404	Slc22a23	3.78779	0.94263	4.0183
Col6a1	126.722	231.88	0.5465	Reln	14.5495	3.37171	4.3152
Rexo2	65.7121	120.237	0.5465	Kcnab1	15.1966	3.5089	4.3309
Rpl23	209.083	379.369	0.5511	Ephx2	77.742	17.7835	4.3716
Mxra8	39.1849	71.0761	0.5513	Gabra1	17.4017	3.94741	4.4084

Nop58	29.5173	53.5054	0.5517	Plp1	20.3197	4.59678	4.4204
Rpl11	254.289	460.673	0.5520	Slc35f1	7.50731	1.5061	4.9846
Itgb5	33.1312	60.0176	0.5520	Erbb3	8.84018	1.70331	5.1900
Csrp1	208.279	377.195	0.5522	Mbp	13.8184	2.24595	6.1526
Zyx	46.2838	83.8168	0.5522	Frzb	38.0721	5.90218	6.4505
Thbs3	21.1978	37.8856	0.5595	St8sia4	4.61317	0.70496	6.5438
Id2	65.4481	116.846	0.5601	Fign	3.53079	0.52287	6.7527
Rpl17	549.64	977.684	0.5622	Rspo3	52.1262	7.38164	7.0616
Kif26b	9.2843	16.4649	0.5639	Ahr	9.73049	1.35532	7.1795
Tgfb2	31.7657	55.9602	0.5676	Ppfia2	10.4974	1.44223	7.2786
Cyp1b1	35.6145	62.4952	0.5699	Ttyh1	9.9399	1.33621	7.4389
Nsa2	55.1097	96.0685	0.5737	Aatk	10.6764	1.10906	9.6265
Prpf38b	35.4097	61.0897	0.5796	Kif5a	12.8872	1.31465	9.8028
Rrbp1	72.8054	125.513	0.5801	Kcna2	4.09267	0.34572	11.8381
Cpe	99.1251	169.641	0.5843	Gpr174	29.0947	2.09188	13.9084
Galnt2	40.6892	69.5177	0.5853	Dpcr1	60.2812	2.05655	29.3118
Ik	70.3244	118.961	0.5912				
Hnrnpa2b1	128.992	217.546	0.5929				

Table S3

Altered pathways in RNA-seq of *Prdm6$^{f/f}$; SM22-Cre* mice DA compared to wildtype littermates based on Gene Ontology (GO) analysis.

Downregulated pathways			
GO biological process complete	upload	raw P-value	FDR
membranous septum morphogenesis (GO:0003149)	3	6.35E-05	2.08E-02
elastic fiber assembly (GO:0048251)	3	1.00E-04	2.86E-02
smooth muscle tissue development (GO:0048745)	4	2.96E-05	1.23E-02
collagen fibril organization (GO:0030199)	7	6.91E-08	1.21E-04
extracellular matrix assembly (GO:0085029)	4	6.35E-05	2.13E-02
integrin-mediated signaling pathway (GO:0007229)	6	1.58E-05	8.89E-03
cell-matrix adhesion (GO:0007160)	6	1.13E-04	3.19E-02
connective tissue development (GO:0061448)	10	1.69E-06	1.66E-03
muscle contraction (GO:0006936)	8	3.21E-05	1.30E-02
regulation of vasculature development (GO:1901342)	9	1.20E-04	3.30E-02
blood vessel morphogenesis (GO:0048514)	13	4.89E-06	4.28E-03
blood vessel development (GO:0001568)	14	9.30E-06	6.10E-03
vasculature development (GO:0001944)	14	1.68E-05	9.10E-03
tube development (GO:0035295)	21	6.31E-07	7.64E-04
developmental process (GO:0032502)	59	6.84E-06	4.90E-03
**Upregulated pathways**			
GO biological process complete	upload	raw P-value	FDR
regulation of establishment of endothelial barrier (GO:1903140)	3	2.91E-04	2.73E-02
regulation of endothelial cell development (GO:1901550)	3	2.91E-04	2.71E-02
outflow tract septum morphogenesis (GO:0003148)	4	5.15E-05	7.31E-03
aorta development (GO:0035904)	5	4.98E-05	7.33E-03
endothelial cell migration (GO:0043542)	5	7.67E-05	1.03E-02
cell-cell adhesion (GO:0098609)	12	1.35E-05	2.40E-03
negative regulation of cell differentiation (GO:0045596)	16	7.48E-06	1.49E-03

Table S4 Pathway analysis of Cluster 10

pathway	pValue	Min FDR	Network Objects from Active Data
DNA damage_ATM/ATR regulation of G2/M checkpoint: cytoplasmic signaling	1.640E-08	9.024E-06	p38alpha (MAPK14), Chk2, Brca1, Histone H3, IPP-2, CDC25C, beta-TrCP, CDC25B, JAB1, PP2A regulatory, Aurora-A, MLCP (reg), Chk1, MEKK1(MAP3K1), PEA15, ATM, B56G, GADD45 alpha, Aurora-B, PLK1, p38 MAPK, JIK
Cell cycle_The metaphase checkpoint	9.167E-07	1.035E-04	ZW10, SPBC24, CENP-H, INCENP, SPBC25, CENP-E, BUB1, Aurora-A, CDCA1, CENP-F, MAD1 (mitotic checkpoint), BUBR1, Aurora-B, HEC, PLK1, AF15q14
Cell cycle progression in Prostate Cancer	3.348E-06	2.354E-04	STAT5A, JAK2, Cyclin B, Tcf(Lef), E2F1, CDC25B, ERK1/2, p21, MNK1, CDK6, STAT3, WNT, AKT(PKB), SP1, p38 MAPK, mTOR
DNA damage_ATM/ATR regulation of G2/M checkpoint: nuclear signaling	6.350E-06	3.729E-04	Chk2, Brca1, Cyclin B, Cyclin B2, CDC25C, GTSE1, Reprimo, CDH1, BTG2, p21, ATRIP, Chk1, ATM, GADD45 alpha, TTK, RBBP8 (CtIP), PLK1
Development_Positive regulation of WNT/Beta-catenin signaling at the receptor level	7.307E-06	3.831E-04	p38alpha (MAPK14), Bcl-9, UBPY, Tcf(Lef), Porcn, CRIPTO, ERK1/2, MKK7 (MAP2K7), IQGAP, RNF43, USP19, p38beta (MAPK11), ZNRF3, LGR6, WNT7A, WNT, Itch, LRP5, DPY19L3, LGR5, Rab8B

Table S5. Primary antibodies used in this study

Name	Manufacturer	Cat#
TFAP2B	CST	#2509
GAPDH	CST	21118S
SOX9	SIGMA	HPA0001759
CD31	BD	550274
Fibronectin	ABCAM	ab6328
Ki67	ABCAM	ab66155
VE-Cadherin	BD	550548
MYH11	ABCAM	ab53219
aSMA	SIGMA	C6198
H4K20me1	ABCAM	ab9051

Table S6. Primers used in this study

Amplicon	Sequence
Gapdh F	AGGTCGGTGTGAACGGATTTG
Gapdh R	TGTAGACCATGTAGTTGAGGTCA
Prdm6 F	TTCTGTCCCCGGAGAAAGTG
Prdm6 R	CTCGACTTACTAGGCTCCCCA
Tfap2b F	GCGTCCCAAGCCATAGCTC
Tfap2b R	ACTCTGGTGGTAGGGTAGCG
Sox9 F	GAGCCGGATCTGAAGAGGGA
Sox9 R	GCTTGACGTGTGGCTTGTTC
Myh11 F	GAGCAAACTCAGGAGAGGAAAC
Myh11 R	GTCCCGAGCGTCCATTTCTTC
Tagln F	CAACAAGGGTCCATCCTACGG
Tagln R	ATCTGGGCGGCCTACATCA
Kdr F	TTTGGCAAATACAACCCTTCAGA
Kdr R	GCAGAAGATACTGTCACCACC
Wnt1 F	GGTTTCTACTACGTTGCTACTGG
Wnt1 R	GGAATCCGTCAACAGGTTCGT
